# Supplementary material for: Stakeholder engagement in a hypertension and diabetes prevention research program: Description and lessons learned
Source: PLoS One. 2022 Oct 20;17(10):e0276478. doi: 10.1371/journal.pone.0276478 (PMC9584412; doi:10.1371/journal.pone.0276478)
Supplement: Attachment — Submitted filename: PONE-D-21-05456_R1_A.pdf [file pone.0276478.s004.pdf]

PLOS ONE

Stakeholder engagement in a hypertension and diabetes prevention research program – Description and Lessons learned

--Manuscript Draft--

**Manuscript Number:**	PONE-D-21-05456R1
**Article Type:**	Research Article
**Full Title:**	Stakeholder engagement in a hypertension and diabetes prevention research program – Description and Lessons learned
**Short Title:**	Stakeholder engagement
**Corresponding Author:**	Archana Shrestha<br>Kathmandu University Hospital: Dhulikhel Hospital<br>Dhulikhel, Bagmati NEPAL
**Keywords:**	Stakeholder engagement; hypertension; diabetes; Workplace settings; Nepal
**Abstract:**	Background<br>Stakeholder engagement is important from the management point of view to capture knowledge, increase ownership, reduce conflict, encourage partnership, as well as to develop an ethical perspective that facilitates inclusive decision making and promotes equity. However, there is dearth of literature in the process of stakeholder engagement. The purpose of this paper is to describe the process of increasing stakeholder engagement and highlight the lessons learnt on stakeholder engagement while designing, implementing and monitoring a study on diabetes and hypertension prevention in workplace settings in Nepal.<br>Methodology<br>We identified the stakeholders based on the 7P framework: Patients and public (clients), providers, payers, policy makers, product makers, principal investigators and purchasers. The identified stakeholders were engaged in prioritization of the research questions, planning data collection, designing, implementing and monitoring the intervention. Stakeholders were engaged through focus group discussions, in-depth interviews, participatory workshops, individual consultation, information sessions and representation in study team and implementation committees.<br>Results<br>The views of the stakeholders were synthesized in each step of the research process, from designing to interpreting the results. Stakeholder engagement helped to shape the methods and plan, and process for participants recruitment and data collection. In addition it enhanced adherence to intervention, mutual learning, and smooth intervention adoption. The major challenges were the time-consuming nature of the process, language barriers, and the differences in health and food beliefs between researchers and stakeholders.<br>Conclusion<br>It was possible to engage and benefit from stakeholder's engagement on the design, implementation and monitoring of a workplace-based hypertension and diabetes management research program in Nepal.
**Order of Authors:**	Archana Shrestha
	Dipesh Tamrakar
	Bhawana Shrestha
	Biraj Man Karmacharya
	Abha Shrestha
	Prajjwal Pyakurel
	Donna Spiegelman
**Response to Reviewers:**	Subject: Re-submission of an original research article to PLOS ONE journal addressing the reviewer's comment<br><br>Dear sir/madam,

Powered by Editorial Manager® and ProduXion Manager® from Aries Systems Corporation

It is our pleasure to re-submit to you and the editorial teams our manuscript entitled "Stakeholder Engagement in a Hypertension and Diabetes Prevention Research Program – Description and Lessons Learned". The paper aims to describe the stakeholder engagement process and the lessons learned from this process while designing, implementing and monitoring a study on diabetes and hypertension prevention in workplace settings in Nepal. It provides important insights for future researchers that aim to engage stakeholders in national-level preventive programs in health.
We would like to thank all the reviewers for their constructive comments. The responses to the reviewer's comments are addressed individually below:

Reviewer 1
This manuscript describes stakeholder engagement activities and lessons learned in diabetes and hypertension prevention study in workplace settings in Nepal. The authors describe stakeholder engagement activities during the formative, intervention development, and study conduct phases of this project. In general, this paper could benefit from a grammatical review as there are several errors throughout. Additionally, the literature review should be updated to reflect current gaps, as there are several existing articles to date that offer engagement implementation examples, which was stated as a gap and strength of current paper. The impact of stakeholder engagement on research activities and translation is an area for further contribution and an opportunity where this manuscript could offer insight. Areas of stakeholder impact should be more clearly identified in the paper. In addition to above, consider the following to strengthen this manuscript:
-Consider re-designing Table 1 into a Figure and move to first presentation of stakeholders using 7P Framework in Methods section (not in Results section as current placement).
>> The Table 1 is converted to figure and shifted to methods section
-Although it was stated that results from qualitative work is under consideration for publication elsewhere, this section could benefit from including more specifics regarding questions asked during focus group sessions/in-depth interviews with stakeholders and/or qualitative supporting quotes from discussions, since these informed intervention. Who came up with interview questions? How did the research team decide which feedback to implement?
>> The result and methods are available in detailed in the paper below and it is now cited.
Tamrakar D, Shrestha A, Rai A, Karmacharya BM, Malik V, Mattei J, et al. Drivers of healthy eating in a workplace in Nepal: a qualitative study. BMJ Open. 2020;10: e031404. doi:10.1136/bmjopen-2019-031404
-Consider creating a new Table 1 in Results that includes stakeholder type and the actual activities that the stakeholders were engaged in (i.e. formative and then subheading for which activity i.e. focus group). It was not clear within text the myriad of stakeholders who participated in the various study processes. This table could also include a column "Impact" to highlight how stakeholder contributions informed the study and in what way(s).
>>Table is generated as per your suggestions

Reviewer 2
An important work in the area stakeholder engagement. Generally, well written but needs some language editing and improvement in flow in some sections.
Abstract
Lines 39-41. What is described does not include interpreting the results and results dissemination.
>> It is revised as per your suggestion
Lines 47: I am not sure increased trust in research and confidence with the researchers is a result or what can be inferred (or assumed) given the processes and outcomes. What evidence do you have for this allusion?
>> It is revised as per your suggestion
Line 54: The manuscript has not reported on engaging the stakeholders in the evaluation may be monitoring.
>>The comment is addressed
Introduction
Line 62: Give reference
>> Reference is added

Powered by Editorial Manager® and ProduXion Manager® from Aries Systems Corporation

See comment on line 67
>> comment addressed
Materials and methods
-Line 88: Aim would be more appropriate than "theme"
>> "theme" is changed to "aim"
-Sub-titles e.g., Phase 1 Formative study would be better be presented as Phase 1: Formative study
-Lines 106 to lines 202: Flow in stakeholder engagement needs to be improved for easier reading. I suggest a main sub-title "Stakeholder engagement" and smaller sub-titles as follows: stakeholder definition, engagement objectives, stakeholder identification, and stakeholder methods of engagement.
>> Its flow is changed according to the suggestion provided.
-Not sure it is necessary to number the phases e.g., line 134
>>The phrases are now un-numbered
-Lines 135-137. What criteria did you use to identify/include the individuals from each stakeholder category after conducting the mapping?. This also applies to those included in focus groups discussion (FDGs).
>> Added to the manuscript. We selected adults aged 18 years or older, employee of the hospital, who provided informed consent in each stakeholder category.
-Purposive selection for interviews- what specific information did you expect that these participants could be having unlike others
>> They had unique experience of making first hand decisions and implementation at the cafeteria and other wellness programs for the employees. These interviewees brought their perspectives on the decision making system, how it affected healthy food choices in the cafeteria and other administrative decisions regarding employee's health. This would provide insights into the facilitators and barriers to healthy eating in the workplace environment.
-I think its important to mention qualitative methods were used for FDGs and the in-depth interviews- as the methods hold specific benefits and give support from the literature on the choice of method
>> >> The result and methods for FGDs and in depth interviews are available in detailed in the paper below and it is now cited.
Tamrakar D, Shrestha A, Rai A, Karmacharya BM, Malik V, Mattei J, et al. Drivers of healthy eating in a workplace in Nepal: a qualitative study. BMJ Open. 2020;10: e031404. doi:10.1136/bmjopen-2019-031404
-Line 172: I think topic rather than theme will be more appropriate
>> The word theme is replaced by the topic
-What was the stakeholders' role in the workshops?
>>The stakeholders provided their inputs in decision making. We presented the initial findings with the stakeholders and collected their feedback in the interpretation of the results.

Results
-Line 211: What informed/guided the inclusion to a certain category (investigator vs. advisor). Any evidence from literature?
>> Investigator were those who directly participated in the day-to-day implementation of the study, compared to advisors who provided their suggestions and guidances when asked for.
-As a reader, I was expecting more details of the FDGs and interviews, but I saw at the end the statement that the results are under consideration for publication elsewhere. Just wondering, the implication of this given that data needs to be availed by authors.
>>>> The result and methods are available in detailed in the paper below and it is now cited.
Tamrakar D, Shrestha A, Rai A, Karmacharya BM, Malik V, Mattei J, et al. Drivers of healthy eating in a workplace in Nepal: a qualitative study. BMJ Open. 2020;10: e031404. doi:10.1136/bmjopen-2019-031404
-Lines 231-232: They suggested to add the healthy items which will demand less time and resources like adding the automated machines such as roti makers. Rephrase I don't think automated machines needs less resource (may be human but not financial)
>> The sentence is rephrased as "They suggested to add the healthy items which will demand less time and human resources like adding the automated machines such as roti makers."
-Interchange order of sentences on lines 218-219 and lines 219-221 for better flow.
>> The sentences are interchanged

	-What was the outcome of the food testing and how did it inform the intervention?<br>>> Added to the manuscript. Participant's perspectives on brown rice was found to be changed after tasting. Participants were willing to switch to brown rice as they found that brown rice had higher quality.[17]<br>-Rephrase lines 356-358 for clarity<br>>>The line is rephrased for clarity<br>-Lines 392-394: There is nowhere that is reported how the stakeholders were involved in interpreting the data.<br>>> The comment is addressed in the manuscript.<br>Discussion<br>Lines 379 to 387: It would be useful if the authors could highlight on how they incorporated the views of the stakeholders in each step, probably in a Table format. For example; What was the final goal and what stakeholder input lead to that outcome.<br>>> We added some ways that we used to incorporate views of stakeholders. (. For example, we formed cafeteria intervention committee based on the suggestion of cafeteria manager in in-depth interview. Brown-rice tasting experiment was also conducted to solve the concern of consumers and chefs on the acceptability of brown rice in workshop.)<br><br>Other comments<br>1.Please ensure you have included the registration number for the clinical trial referenced in the manuscript.<br>The registration number is added in the manuscript. This study was registered on clincaltrials.gov (Identification Member:NCT03447340; Registration date: 27th February, 2018).<br>2.To help ensure that the wording of your manuscript is suitable for publication, would you please also add this statement at the beginning of the Methods section of your manuscript file.<br>The ethical statement is added in the publication.<br>Ethical consideration: We received approval from the institutional review board of Kathmandu University School of Medical Sciences and Nepal Health Research Council for the human subject contacts and obtained written informed consent from the participants before collecting data for research purpose.<br>3.Data availability statement.<br>The dataset is uploaded is OSF. The link to dataset is https://osf.io/bqsrd/<br><br>I think it is important for the reader to know where the project is at the moment- is still being implemented and prospect of sustainability given that its one of the objective of involving stakeholders<br>We look forward to your comments and remain with best regards;
**Additional Information:**	
**Question**	**Response**
**Financial Disclosure**<br><br>Enter a financial disclosure statement that describes the sources of funding for the work included in this submission. Review the submission guidelines for detailed requirements. View published research articles from *PLOS ONE* for specific examples.<br><br>This statement is required for submission and **will appear in the published article** if the submission is accepted. Please make sure it is accurate.	•DS, AS, DT<br>•Award no: 5DP1ES02545903<br>•National Institute of Health<br>•https://www.nih.gov/about-nih<br>•The funders had no role in study design, data collection and analysis, decision to publish, or preparation of the manuscript.

**Unfunded studies**<br>Enter: *The author(s) received no specific funding for this work.*<br><br>**Funded studies**<br>Enter a statement with the following details:<br>• Initials of the authors who received each award<br>• Grant numbers awarded to each author<br>• The full name of each funder<br>• URL of each funder website<br>• Did the sponsors or funders play any role in the study design, data collection and analysis, decision to publish, or preparation of the manuscript?<br>• **NO** - Include this sentence at the end of your statement: *The funders had no role in study design, data collection and analysis, decision to publish, or preparation of the manuscript.*<br>• **YES** - Specify the role(s) played.<br><br>* typeset	
**Competing Interests**<br><br>Use the instructions below to enter a competing interest statement for this submission. On behalf of all authors, disclose any competing interests that could be perceived to bias this work—acknowledging all financial support and any other relevant financial or non-financial competing interests.<br><br>This statement is required for submission and **will appear in the published article** if the submission is accepted. Please make sure it is accurate and that any funding sources listed in your Funding Information later in the submission form are also declared in your Financial Disclosure statement.<br><br>View published research articles from *PLOS ONE* for specific examples.	The authors have declared that no competing interests exist.

Powered by Editorial Manager® and ProduXion Manager® from Aries Systems Corporation

**NO authors have competing interests**<br><br>Enter: *The authors have declared that no competing interests exist.*<br><br>**Authors with competing interests**<br><br>Enter competing interest details beginning with this statement:<br><br>*I have read the journal's policy and the authors of this manuscript have the following competing interests: [insert competing interests here]*<br><br>* typeset	
**Ethics Statement**<br><br>Enter an ethics statement for this submission. This statement is required if the study involved:<br><br>• Human participants<br>• Human specimens or tissue<br>• Vertebrate animals or cephalopods<br>• Vertebrate embryos or tissues<br>• Field research<br><br>Write "N/A" if the submission does not require an ethics statement.<br><br>General guidance is provided below. Consult the submission guidelines for detailed instructions. **Make sure that all information entered here is included in the Methods section of the manuscript.**	We received approval from the institutional review board of Kathmandu University School of Medical Sciences and Nepal Health Research Council for the human subject contacts and obtained written informed consent from the participants before collecting data for research purpose.

Powered by Editorial Manager® and ProduXion Manager® from Aries Systems Corporation

**Format for specific study types**<br><br>**Human Subject Research (involving human participants and/or tissue)**<br>• Give the name of the institutional review board or ethics committee that approved the study<br>• Include the approval number and/or a statement indicating approval of this research<br>• Indicate the form of consent obtained (written/oral) or the reason that consent was not obtained (e.g. the data were analyzed anonymously)<br><br>**Animal Research (involving vertebrate animals, embryos or tissues)**<br>• Provide the name of the Institutional Animal Care and Use Committee (IACUC) or other relevant ethics board that reviewed the study protocol, and indicate whether they approved this research or granted a formal waiver of ethical approval<br>• Include an approval number if one was obtained<br>• If the study involved *non-human primates*, add *additional details* about animal welfare and steps taken to ameliorate suffering<br>• If anesthesia, euthanasia, or any kind of animal sacrifice is part of the study, include briefly which substances and/or methods were applied<br><br>**Field Research**<br><br>Include the following details if this study involves the collection of plant, animal, or other materials from a natural setting:<br>• Field permit number<br>• Name of the institution or relevant body that granted permission	
**Data Availability**<br><br>Authors are required to make all data underlying the findings described fully available, without restriction, and from the time of publication. PLOS allows rare exceptions to address legal and ethical concerns. See the PLOS Data Policy and FAQ for detailed information.	Yes - all data are fully available without restriction

A Data Availability Statement describing where the data can be found is required at submission. Your answers to this question constitute the Data Availability Statement and **will be published in the article**, if accepted.<br><br>**Important:** Stating 'data available on request from the author' is not sufficient. If your data are only available upon request, select 'No' for the first question and explain your exceptional situation in the text box.<br><br>Do the authors confirm that all data underlying the findings described in their manuscript are fully available without restriction?	
**Describe where the data may be found in full sentences. If you are copying our sample text, replace any instances of XXX with the appropriate details.**<br><br>• If the data are **held or will be held in a public repository**, include URLs, accession numbers or DOIs. If this information will only be available after acceptance, indicate this by ticking the box below. For example: *All XXX files are available from the XXX database (accession number(s) XXX, XXX.*).<br>• If the data are all contained **within the manuscript and/or Supporting Information files**, enter the following: *All relevant data are within the manuscript and its Supporting Information files.*<br>• If neither of these applies but you are able to provide **details of access elsewhere**, with or without limitations, please do so. For example:<br><br>*Data cannot be shared publicly because of [XXX]. Data are available from the XXX Institutional Data Access / Ethics Committee (contact via XXX) for researchers who meet the criteria for access to confidential data.*<br><br>*The data underlying the results presented in the study are available from (include the name of the third party*	The data has been deposited to Open Science Framework (OSF) public repository with URL: https://osf.io/bqsrd/

and contact information or URL).<br>• This text is appropriate if the data are owned by a third party and authors do not have permission to share the data.<br><br>* typeset	
Additional data availability information:	

Manuscript Click here to access/download;Manuscript;2 Stakeholder Engagement_Manuscript.docx

Stakeholder engagement in a hypertension and diabetes prevention research program – Description and Lessons learned

Archana Shrestha[1,2*], Dipesh Tamrakar[1], Bhawana Shrestha[1], Biraj Man Karmacharya[1], Abha Shrestha[1], Prajjwal Pyakurel[2], Donna Spiegelman[3]

[1] Department of Public Health, Kathmandu University School of Medical Sciences, Dhulikhel, Nepal

[2] Department of Community Medicine, BP Koirala Institute of Health Sciences, Dharan, Nepal

[3] Department of Biostatistics, Yale School of Public Health, New Haven, USA

* Corresponding author: Dr. Archana Shrestha; E-mail: deararchana@gmail.com

Abstract

Background

Stakeholder engagement is important from the management point of view to capture knowledge, increase ownership, reduce conflict, encourage partnership, as well as to develop an ethical perspective that facilitates inclusive decision making and promotes equity. However, there is dearth of literature in the process of stakeholder engagement. The purpose of this paper is to describe the process of increasing stakeholder engagement and highlight the lessons learnt on stakeholder engagement while designing, implementing and monitoring a study on diabetes and hypertension prevention in workplace settings in Nepal.

Methodology

We identified the stakeholders based on the 7P framework: Patients and public (clients), providers, payers, policy makers, product makers, principal investigators and purchasers. The identified stakeholders were engaged in prioritization of the research questions, planning data collection, designing, implementing and monitoring the intervention. Stakeholders were engaged through focus group discussions, in-depth interviews, participatory workshops, individual consultation, information sessions and representation in study team and implementation committees.

Results

The views of the stakeholders were synthesized in each step of the research process, from designing to interpreting the results. Stakeholder engagement helped to shape the methods and plan, and process for participants recruitment and data collection. In addition it enhanced adherence to intervention, mutual learning, and smooth intervention adoption. The major

challenges were the time-consuming nature of the process, language barriers, and the differences in health and food beliefs between researchers and stakeholders.

Conclusion

It was possible to engage and benefit from stakeholder's engagement on the design, implementation and monitoring of a workplace-based hypertension and diabetes management research program in Nepal.

INTRODUCTION

New scientific evidence is produced in great volume [1]. However, much of this evidence has been difficult to implement in practice[2]. Stakeholder engagement can help to address this need by improving the relevance of research by increasing its transparency and accelerating the adoption into real world setting[3]. Stakeholder engagement is also important to generate knowledge, increase ownership, reduce conflict, encourage partnership, facilitate an inclusive decision making and promote equity[4].

Stakeholder engagement is promoted by health research funding organizations as well as researchers to achieve the desired goal[5]. Stakeholders can be engaged across stages of research including identifying topics, choosing hypotheses, analyzing data, and disseminating findings[6,7]. The levels of involvement range from consultation, to collaboration in bi-directional partnerships and to leading research projects[8].

Despite considerable attention to stakeholder engagement, there is limited information on how stakeholders are engaged. Some reviews describe the engagement in published literature and report impacts of engagement [9–12]. However, there is lack of reporting on how an engagement is implemented. It has been recommended that researchers systematically document and report information about engagement and its impact on individual projects[9,13]. Therefore, there is the need for demonstrating approaches that have been used to stakeholder engagement and opportunities to learn more about engagement in individual research projects. The purpose of this paper is to describe the process of stakeholder engagement and the lessons learned from this process while designing, implementing and monitoring a study on diabetes and hypertension prevention in workplace settings in Nepal.

MATERIALS AND METHODS

Study setting

Nepal Pioneer Worksite Intervention Study [14] started in 2016 at Dhulikhel Hospital-Kathmandu University Hospital in central Nepal with a goal to prevent diabetes and hypertension among the employees of Dhulikhel Hospital. Dhulikhel Hospital is an independently owned, not-for-profit institution which was conceived and supported by the Dhulikhel community. This study was registered on clincaltrials.gov (Identification Member:NCT03447340; Registration date: 27th February, 2018). The study was planned and executed in three phases:

Phase-1: Formative study

We conducted a formative study to gather data useful to develop and implement an intervention to promote healthy eating at the worksite. The major aim of the formative study was appropriateness to culture and geography to develop meaningful interventions at individual and organizational level. The objective was to define and assess attributes of the study participants, understand the context, tailor intervention at local context, and build relationship between researcher, study populations and other stakeholders.

Phase-2: Develop culturally appropriate intervention and define research attributes

In the second phase, we used the information from the formative study to develop a culturally appropriate environmental level intervention to promote healthy eating in the cafeteria of the workplace. We further used this information to define study attributes such as primary outcomes, study design, implementation and monitoring strategies, and analysis plan.

Phase-3: Test the effectiveness of the intervention

In the third phase, we tested the effectiveness of the environmental level intervention using a two-step intervention study. In the first step, we assessed the effectiveness of a 6-month cafeteria intervention on cardio-metabolic risk using a pre-post design. In the second step, we conducted a 6-month, open-masked, two-arm randomized trial by allocating half of the participants to an individual behavioral intervention for the prevention of cardio-metabolic risk in order to estimate the additional effect of an individual level intervention. The design of the study has been published elsewhere[14].

Stakeholder engagement

Stakeholder definition

We defined stakeholder as an individual or group affected by or who can influence the environmental intervention to promote healthy eating in the workplace.

Engagement objectives

Our objectives of stakeholder engagement were (a) to identify the specific research needs in their worksite for promoting healthy eating, (b) to develop culturally appropriate healthy eating intervention; (c) to increase stakeholders' participation in study process such as determining study objectives and methods, describing study intervention, recruiting participants, implementing and monitoring the healthy eating intervention; (d) interpreting study results; and (e) enhancing sustainability.

Identification of stakeholders

We adopted the 7Ps framework[15] that identifies key groups to consider for engagement. The first, **patients and the public**, represented the current and potential consumers of patient-centered health care and population-focused public health. The second were **providers**, including individuals and organizations that provide care to patients and populations. **Purchasers**, the individuals and entities responsible for underwriting the costs of health care, such as employers, made up the third group. The fourth group consisted of **payers** who were responsible for reimbursement of medical care, such as insurers. The fifth composed of public **policy makers** and policy advocates working in the non-governmental sector. **Product makers**, representing drug and device manufacturers, comprised the sixth group, and **principal investigators,** or other researchers, made up the seventh. Since this was not a typical patient focus study, but rather a community-based prevention trial in an institutional setting, we modified the definition of the 7Ps according to our context. (Figure 1)

Figure 1: Stakeholder mapping for the Nepal Pioneer Worksite Intervention study to prevent cardiometabolic risk

Stakeholder engagement methods

We involved stakeholders in all three phases.

At formative study phase

Formation of study team: After mapping out the stakeholder for the study, individuals representing each stakeholder category were approached individually in-person and the study team was formed. We selected adult aged 18 years or older, employee of the hospital, who provided informed consent as stakeholder in each category. The stakeholders represented as co-investigators and advisors.

Focus Group Discussions: We conducted four focus group discussions with 33 employees of the hospital to explore the current healthy eating environment, understand drivers and barriers for eating healthy, and to obtain their recommendations on improving environment to promote healthy eating at the hospital cafeteria.

The focus groups were conducted within the workplace setting in a private room to ensure confidentiality and open sharing of opinion. The FGDs were conducted in Nepali and audio taped after obtaining informed consent from the participants. The investigators, AS, or DT, moderated all FGDs in Nepali language and were assisted by a note-taker. In each session, a brief introduction of the study and ethical considerations about maintaining confidentiality of the participants, were explained. The moderator asked open-ended questions and probed for detail information. We used iterative process by discussing each FGD immediately after completion and suggesting necessary detail probing in emerging themes from finding.

In-depth interviews: We conducted 9 in-depth interviews with purposively selected hospital employees including a finance manager, a cafeteria manager, an administrative

manager and 6 cafeteria operators after receiving informed consent. The investigators DT or AS conducted a semi-structured in-depth Interviews in Nepali language using a pretested interview guide in private room of the hospital. We used the iterative process for data collection. After each interview, we discussed each interview and identified the topics to be deeply explored into themes emerging in earlier interviews. All FGDs and interviews were transcribed verbatim into Nepali by trained research assistants. We used thematic analysis approach [16] to analyze the data.

At development of research design and intervention phase

Workshops: We conducted three iterative workshops with the representation of hospital administration, cafeteria managers, cafeteria staff, nutrition department, employee and researchers. Each workshop was facilitated by DT, a co-investigator and an employee, using a workshop schedule and discussion guideline. The workshops took 3 hours. In the first hour, the facilitator explained the link between healthy eating and cardiometabolic risks. In the second hour, the findings from the formative study were shared in an interactive session, and in the third hour a guided discussion with the participants was conducted. The themes of the guided discussion were: components of the healthy eating interventions, mode of delivery, mode of implementation and monitoring, and method of evaluation - that led to design of the effectiveness study. The stakeholders' in workshops provided inputs in decision making. We presented the initial findings with the stakeholders and collected their feedback in the interpretation of the results.

Food taste experiments: A major theme that emerged in focus group discussions, in-depth interviews and workshop was regarding acceptability of introducing whole grains in the cafeteria, especially brown rice. So, we conducted a brown rice tasting experiment with 40 employees of the hospital. Participant's perspectives on brown rice was found to be changed after tasting. Participants were willing to switch to brown rice as they found that brown rice had higher quality.[17]

c) Individual consultation: The results of the formative study with further action plan were discussed individually with the hospital administrator, cafeteria manager, cafeteria chefs, nutritionist of the hospital and few employees of the hospital to get further feedback.

At testing of intervention phase

Meeting of study team: The study team met online every week for 30 to 60 minutes to discuss on a predetermined agenda and all decisions were taken jointly and recorded in written.

Information sessions: A 20-minute information session was conducted in all departments of the hospital to provide information regarding the study and collecting feedback. About 22 sessions were conducted with participation of 4 to 20 employees in each session. In the first five minutes, the study design was explained, the next 5 minutes were dedicated for the call for participation and expectations from the participants, and 10 minutes for discussion and feedback.

Formation of cafeteria intervention committee: A canteen intervention committee was formed the representation ~~of~~ hospital administrator, human resource department, finance department, hygiene monitoring department, non-communicable disease prevention and management department, nutrition department, canteen managers, employee and

researchers. This committee steered the development, implementation and monitoring of the cafeteria intervention. The committee made the plans for the cafeteria intervention, implementation and monitoring. It meets once a month to discuss about the progress, problems and solutions.

Ethical consideration

We received approval from the institutional review board of Kathmandu University School of Medical Sciences and Nepal Health Research Council for the human subject contacts and obtained written informed consent from the participants before collecting data for research purpose.

RESULTS

Stakeholder identification

Based on the 7P framework, the identified stakeholders are presented in Figure 1. The identified stakeholders were- customers or employees (patient and the public; purchasers); cafeteria managers, chef and staff (providers); hospital administration and finance department (payers; policy makers); Human resource department (policy maker); Cafeteria employee, bakery and Rice mills (Product maker); and Research team members (principal investigators).

Outcome of stakeholder engagement

Table 1 presents the stakeholder engagement in different activities within different study phases

Table 1: Stakeholder engagement in different activities within different study phases

**Study phase**	**Activity**	**Stakeholder involved**
**During Formative phase**	Formation of study team	• Principal investigator (Research team members)<br>• Purchasers (Employees including a medical doctor, a laboratory personnel, a pharmacist and a community programs expert)<br>• Payers (The Hospital administrator)<br>• Policy maker (The Hospital administrator, A nutritionist)<br>• Provider (A cafeteria manager)
	Focus Group Discussions	• Principal investigator (Research team members)<br>• Purchasers (Employees))
	In-depth interviews	• Provider (Cafeteria operator, Cafeteria manager)<br>• Product maker (Cafeteria Staffs, Bakery and Rice mills)<br>• Policy maker (Human resource manager)
**During development of research**	Workshops	• Principal investigator (Research team members)<br>• Provider (Cafeteria operator, Cafeteria manager)<br>• Product maker (Cafeteria Staff[illegible]<br>• Purchasers (Employees)

**design and intervention phase**	Individual consultation	• Principal investigator (Research team members)<br>• Purchasers (Employees including medical doctor, laboratory personnel, pharmacist and community programs expert)<br>• Payers (Hospital administrator)<br>• Policy maker (Hospital administrator, Nutritionist)<br>• Provider (Cafeteria operator, Cafeteria manager)
**During testing of intervention phase**	Meeting of study team	• Principal investigator (Research team members)<br>• Purchasers(Employees including a medical doctor, a laboratory personnel, a pharmacist and a community programs expert)<br>• Payers (Hospital administrator)<br>• Policy maker (Hospital administrator, Nutritionist)<br>• Provider (Cafeteria operator, Cafeteria manager)
	Information sessions	• Principal investigator (Research team members)<br>• Policy makers (Departmental head and supervisors)<br>• Purchasers (Employees)
	Formation of cafeteria intervention committee:	• Product maker (Chef, Canteen operator)<br>• Policy makers (Administrative staffs, Nutritionist)<br>• Purchasers(employees including Medical doctors)

During formative study phase

Formation of study team: We formed a study team representing stakeholders from each category as investigator or an advisor. Investigator were those who directly participated in the day-to-day implementation of the study, compared to advisors who provided their suggestion and guidance when asked for [18]. Four employees - a medical doctor, a laboratory personnel, a pharmacist and a community programs expert - joined the team as co-investigators. They brought their scientific expertise and unique perspective as a customer of cafeteria. The hospital administrator, a cafeteria manager and a nutritionist joined as advisor.

Focus Group Discussions: Majority of participants considered that the foods that were available in the hospital cafeteria were healthy. However, items such as confectionaries like cream donut, oily curry and meat, soda drinks, instant noodles etc. were considered unhealthy food. They emphasized that the foods were hygienically prepared and cheaper compared to hotels outside. Participants highlighted that the changes to improve food quality will be ~~well received~~ positively because most of them are health professionals. The most reported factors that would facilitate healthy eating were: (a) addition of healthy food options, (b) replacement of unhealthy foods with healthy options, and (c) appealing presentation of healthy foods. Active involvement of canteen management and administrator in the process of change was highly emphasized. The participants suggested to advertise healthy food options and educate both cafeteria operators and consumers on healthy eating. The major barriers to healthy eating were: (a) unavailability of healthy options, (b) lack of human resources to prepare healthy food, and (c) high price of healthy food and (d) food preferences. They suggested to add the healthy items which will demand less time and resources like adding the automated machines such as roti makers. The support staff commented that they might not be consuming the fruits even if they are added because of the high cost. However, health professionals expressed their willingness to pay more for access to healthy food. The participants were concerned that it would be difficult to change food preferences as the consumers prefer spicy and oily foods. Some of the unhealthy foods are greatly loved such as instant noodles, samosas, cream donuts, soda drinks etc., and changing food habit is challenging.

In-depth interviews: Cafeteria operator commented that the higher-level authorities should be involved in making healthy changes, deciding the menu and fixing the price. One of the

canteen managers highlighted the need of a committee involving the canteen operator, administrative staff, medical doctors and nutritionist to decide on the change in menu, price, and to monitor the availability of healthy options in canteen. The canteen operators mentioned that the hospital cafeteria provides food in subsidized price and does not intend to make profit. The operators were concerned about the lack of knowledge on healthy eating among the cafeteria staff and pointed that providing health education to them could facilitate making changes. They mentioned that the cafeteria staff receive training on hygiene occasionally but have never received training on healthy eating and healthy cooking. The major barrier for healthy eating reported by canteen operator were: (a) lack of adequate human resource to add food options; (b) lack of knowledge on the healthy cooking among cafeteria staff, (c) unavailability of healthy food options in the cafeteria and (d) no food supply for healthy foods such as brown rice, brown bread, organic vegetables etc. The canteen operator reported that they are not well-staffed to provide healthy foods such as whole wheat pan bread (roti), fruits and salad as their preparation is labor intensive. The human resource manager thought that increasing efficiency of the available staff was more important than adding staff. The stakeholders contributed to the interpretation of qualitative data and contextualize the findings. The details of the FGD and Interview results are available elsewhere [19].

During development of research design and intervention phase

Workshops: The discussions in the workshop were very interactive and productive. The cafeteria managers and cafeteria staffs emphasized the need for active involvement of the departments of finance and administration because these departments had major role

deciding the menu and price of the food served in the cafeteria. This led to formation of canteen intervention committee with chairmanship of the hospital administrator and representatives from finance, human resource, nutrition, employee and researchers. There was univocal agreement in the need of adding healthy food items. However, lack of human resource in the cafeteria was a major challenge. In response, automated roti (whole wheat pan bread) maker was suggested. The details on which food to add and which to remove were discussed and finalized in the workshop. In addition, the pre-post design was suggested in contrast to randomized trial to test the effectiveness of the changes in cafeteria on cardiometabolic risk because it would be unethical to provide healthy food items in some cafeterias only. Additionally, the risk of contamination was emphasized as customers do not exclusively go to one cafeteria. Consumers and chefs raised the concern on acceptability of brown rice in place of white rice. Hence, a brown rice tasting experiment was suggested.

Individual consultation: Based on the findings of the FGDs, in-depth interviews and workshops, the study team prepared the description of the cafeteria intervention and proposed study design to test the effectiveness of the intervention on cardiometabolic risks. The proposal was described and discussed in-person with the stakeholders. Lack of comparison group was indicated as a weakness of the study in the initial pre-post design. As a result, six months of control period was added before intervention was implemented to be able to compare outcome measures with and without the intervention.

During testing of intervention phase

Meeting of study team: In the weekly meeting of the study team, the implementation strategies were discussed and finalized in minute details. This included details on

disseminating information on study, call for participation, informed consent administration, data collection from the participants, enrolment planning, and intervention start dates. The internal insights of employees in the study team members guided the decision making. When the effectiveness study started, the progress on enrollment, problems and strategies were discussed weekly. For example, when there was slow blood sample collection, the representative from the laboratory suggested to provide breakfast voucher to the participants. Adding breakfast voucher worth 70 cents in subsequent blood draw appointment speeded up the sample collection. Similarly, it was difficult to enroll the nurses who had night duties. The doctor in the study team suggested the nurse's duty roster and suggested to contact them immediately after their duty is over in the morning. This strategy helped to increase the participation of the nurses in the study.

Information sessions: The information session helped to reach and interact with wider employee group. The department head provided date and venue for the information session. During the information session, feedback was obtained on the design of the study and participant recruitment. A major issue that came up was the involvement of supervisors in participants' recruitment. The employees commented that presence of supervisors during the information session might be perceived as encouragement from them to participate. To make the participation voluntary, we conducted the sessions in absence of higher-level staff in each department, the identifiers of the participants were collected separately and were not disclosed to anyone.

Formation of cafeteria intervention committee: The cafeteria intervention committee had key decisive role in designing, implementing, monitoring and evaluating the cafeteria intervention. Each component of the intervention was discussed and decided by the

intervention committee after weighing their benefits, financial implications and perceived acceptance by the employee. The committee developed a monitoring sheet that the nutritionist and a research team member filled every week to measure the adherence to the defined intervention in all four cafeterias. The monitoring team also interacted with the chefs, cafeteria staffs and customers informally and collected their concerns. The monitoring data were assessed and concerns were presented to the committee every month. The committee discussed the problems and strategies to address. For example, in the initial week, there was problem of supply of whole grains such as brown rice and brown bread. The committee approached the local mill and a local bakery and ordered brown rice and brown bread for the cafeteria. After adding fruits on the menu, the sales of fruits in the first two months were low. The committee decided to launch a week long campaign to promote fruits in each cafeteria which boosted their sales.

Benefits and challenges of stakeholder engagement

We are confident that our project, the project goals, methods and intervention have evolved positively based on our collaborations with stakeholders. Most importantly, their input was necessary in determining and describing cafeteria intervention. Their continuous involvement in monitoring helped in solving problems promptly and enhanced adherence to intervention. In addition, their input shaped methods, plans and processes for recruiting participants and data collection.

While we had a positive experience of involving stakeholder, it was not devoid of challenges. A major challenge was time availability of stakeholders as they were busy in their respective works.

To address this, we scheduled meetings according to the availability of the stakeholders. We had to conduct multiple workshops and information sessions to accommodate the time of cafeteria staff and hospital staff. This was time and resource consuming. It was also important to allow time for stakeholders to get acquainted with their new roles associated with intervention. Their knowledge of a healthy diet was dominated by the cultural norms and there was some misconception. So, we invested time and resources in training them on healthy eating. This also led to initiation of positive changes in cafeterias during the control period before the predetermined intervention date. We also felt that research priorities do not necessarily meet the priorities of the stakeholders. For example, the cafeteria staff emphasized more on the changes in physical structures such as adding culinary. There was language barrier, as researchers were comfortable using scientific jargons such as ‘epidemiology’ ‘control’ ‘confounding’, ‘subjects’ etc. making the stakeholders confused at times. It was difficult to translate or interpret these works well in local language. Additionally, the food beliefs of researchers and stakeholders were different. Stakeholders strongly believe in eating warm ‘white rice’ twice a day. In addition, fruits were not considered appropriate to take during lunch. Knowledge sessions on healthy diets addressed these deep rooted beliefs to some extent but changing food belief system takes longer time and more effort.

DISCUSSION

We identified employees, cafeteria managers, workplace administrators, finance manager, human resource manager, cafeteria staff and researchers as major stakeholders in the Nepal pioneer worksite intervention study to prevent cardiometabolic risks. The stakeholders were included in the study team and they brought their expertise in the subject matter as well as insights as employees and customers of the cafeteria. The exploration of the facilitators and barriers to healthy

eating at their worksite provided a base for defining the intervention and study attributes. Interactive workshops provided opportunity and mandate for the stakeholders to participate in decision making and strengthened the investigator-stakeholder relationship. Individual consultations helped get stronger buy-in and commitment from the higher authorities and employees. Regular meeting of the study team and canteen intervention committee guaranteed continuous engagement in problem solving and decision making. It reinforced ownership and enhanced project sustainability. The major challenges were the time-consuming nature of the process, language barriers, and differences in health and food beliefs between researchers and stakeholders.

We mapped and engaged 7 different types of stakeholders in our study. Patient-centered outcome research projects have reported engaging clinicians, caregivers, advocacy organizations and health system representatives; but only few engaged payers and policy makers[13,20]. We incorporated the views of multiple types of stakeholders into our study during formative phase, development of intervention and study and execution of the study. For example, we formed cafeteria intervention committee based on the suggestion of cafeteria manager in in-depth interview. Brown-rice tasting experiment was also conducted to solve the concern of consumers and chefs on the acceptability of brown rice in workshop. Engagement has most frequently been reported in developing research questions[9]. This study described the effort, attention, resources and flexibility for concerted engagement emphasizing on meaningful and continuous collaboration, shared leadership and decision-making power, and adaptation to the needs of stakeholders. Other researchers have highlighted the importance of sharing power for successful research engagement[21].

The input from different stakeholder influenced our study goals, intervention, methods and materials. This enhanced the appropriateness and relevance in the local context. Similar experience

was reported by other investigators[6]. Making research more beneficiaries-centric, culturally relevant and accessible to potential research participants have been reported to refine proposals, recruitment materials, and participant compensation[22]. In our project, the stakeholders contributed to the interpretation of qualitative data and contextualize the findings, which was also reported by other researchers[11]. This is consistent with the available evidence on positive impacts of engaged research[6,12].

The stakeholder engagement has contributed positively in our study from formative phase to execution. Various levels of stakeholder involvement, such as consultation, collaboration or user-control has been proposed[23]. However, caution has been recommended against assuming that involving higher levels is better[24]. Some researchers in a national survey from UK claimed that decisions about the appropriateness of consumer involvement in research should be made on a case-by-case basis [13].

The major challenges that we faced were the time-consuming nature of the process, language barriers, and differences in health and food beliefs between researchers and stakeholders. Similar challenges have been reported in other studies[6,25]. Lack of time—both for stakeholders and researchers—was the most frequently noted challenge and was identified as a top barrier to engagement in a recent survey of investigators[25]. Thus, it is crucial to develop strategies that maximize stakeholder input with minimum time and resources[26].

The generalizability of our findings is limited because of the unique setting where the research was conducted. Because it was a university hospital, we had an advantage of including the employees with specific research expertise as co-investigators. The stakeholders had more interest in and experience with the health interventions and research. Thus, their knowledge and attitude about engagement may be more positive.

CONCLUSION

It was possible to engage and benefit from stakeholder's engagement on the design, implementation and monitoring of a workplace-based hypertension and diabetes management research program in Nepal. We recommend ongoing program development and implementation by stakeholder input. In future, we plan to evaluate the influence of stakeholders on the research project and assess the engagement and effectiveness through their perspective, as well as longer term outcomes on health decision making beyond the conduction of research.

ACKNOWLEDGMENTS

We would like to acknowledge the Dhulikhel Hospital – Kathmandu University Hospital for their permission and cooperation to conduct this study. We appreciate the contribution of all the study participants.

AUTHOR CONTRIBUTIONS

Conceptualization: Archana Shrestha (AS), Dipesh Tamrakar(DT), Donna Spiegelman (DS)

Formal analysis: Bhawana Shrestha (BS), Biraj Man Karmacharya(BMK), Abha Shrestha (AbS), Prajjwal Pyakurel(PP)

Writing – original draft: Archana Shrestha (AS)

Writing – review & editing: Archana Shrestha (AS), Dipesh Tamrakar (DT), Bhawana Shrestha (BS), Biraj Man Karmacharya(BMK), Abha Shrestha(AbS), Prajjwal Pyakurel(PP), Donna Spiegelman (DS)

REFERENCES

1. Joyce MC. How will I ever keep up with pharmacy changes? Pharmacy Today. 2015;21: 1.

2. Zilberberg MD. The clinical research enterprise: time to change course? Jama. 2011;305: 604–605.

3. Ray KN, Miller E. Strengthening stakeholder-engaged research and research on stakeholder engagement. Journal of comparative effectiveness research. 2017;6: 375–389.

4. Mathur VN, Price ADF, Austin S. Conceptualizing stakeholder engagement in the context of sustainability and its assessment. Construction Management and Economics. 2008;26: 601–609. doi:10.1080/01446190802061233

5. Kok MO, Gyapong JO, Wolffers I, Ofori-Adjei D, Ruitenberg J. Which health research gets used and why? An empirical analysis of 30 cases. Health research policy and systems. 2016;14: 1–18.

6. Shippee ND, Domecq Garces JP, Prutsky Lopez GJ, Wang Z, Elraiyah TA, Nabhan M, et al. Patient and service user engagement in research: a systematic review and synthesized framework. Health Expectations. 2015;18: 1151–1166.

7. Mullins CD, Abdulhalim AM, Lavallee DC. Continuous patient engagement in comparative effectiveness research. Jama. 2012;307: 1587–1588.

8. Lowes L, Hulatt I. Involving service users in health and social care research. Routledge; 2013.

9. Concannon TW, Fuster M, Saunders T, Patel K, Wong JB, Leslie LK, et al. A systematic review of stakeholder engagement in comparative effectiveness and patient-centered outcomes research. Journal of general internal medicine. 2014;29: 1692–1701.

10. Forsythe LP, Szydlowski V, Murad MH, Ip S, Wang Z, Elraiyah TA, et al. A systematic review of approaches for engaging patients for research on rare diseases. Journal of general internal medicine. 2014;29: 788–800.

11. Boote J, Baird W, Sutton A. Involving the public in systematic reviews: a narrative review of organizational approaches and eight case examples. Journal of comparative effectiveness research. 2012;1: 409–420.

12. Brett J, Staniszewska S, Mockford C, Herron- Marx S, Hughes J, Tysall C, et al. Mapping the impact of patient and public involvement on health and social care research: a systematic review. Health expectations. 2014;17: 637–650.

13. Barber R, Boote JD, Cooper CL. Involving consumers successfully in NHS research: a national survey. Health Expectations. 2007;10: 380–391.

14. Shrestha A, Tamrakar D, Karmacharya BM, Shrestha A, Shrestha R, Bhatta RD, et al. Nepal Pioneer Worksite Intervention Study to lower cardio-metabolic risk factors: design and protocol. BMC cardiovascular disorders. 2019;19: 1–10.

15. Concannon TW, Meissner P, Grunbaum JA, McElwee N, Guise J-M, Santa J, et al. A new taxonomy for stakeholder engagement in patient-centered outcomes research. Journal of general internal medicine. 2012;27: 985–991.

16. Boyatzis RE. Transforming qualitative information: Thematic analysis and code development. sage; 1998.

17. Gyawali P, Tamrakar D, Shrestha A, Shrestha H, Karmacharya S, Bhattarai S, et al. Consumer acceptance and preference for brown rice—A mixed- method qualitative study from Nepal. Food Science & Nutrition. 2022; fsn3.2803. doi:10.1002/fsn3.2803

18. Sciences NA of, Science NA of E (US) and I of M (US) C on, Engineering, Policy and P. ADVISING AND MENTORING. On Being a Scientist: A Guide to Responsible Conduct in Research: Third Edition. National Academies Press (US); 2009. Available: https://www.ncbi.nlm.nih.gov/books/NBK214555/

19. Tamrakar D, Shrestha A, Rai A, Karmacharya BM, Malik V, Mattei J, et al. Drivers of healthy eating in a workplace in Nepal: a qualitative study. BMJ Open. 2020;10: e031404. doi:10.1136/bmjopen-2019-031404

20. Forsythe LP, Ellis LE, Edmundson L, Sabharwal R, Rein A, Konopka K, et al. Patient and stakeholder engagement in the PCORI pilot projects: description and lessons learned. Journal of general internal medicine. 2016;31: 13–21.

21. Westfall JM, Fagnan LJ, Handley M, Salsberg J, McGinnis P, Zittleman LK, et al. Practice-based research is community engagement. The Journal of the American Board of Family Medicine. 2009;22: 423–427.

22. Joosten YA, Israel TL, Williams NA, Boone LR, Schlundt DG, Mouton CP, et al. Community engagement studios: a structured approach to obtaining meaningful input from stakeholders to inform research. Academic Medicine. 2015;90: 1646.

23. Dent H. Involving the Public in NHS, Public Health and Social Care Research: Briefing notes for researchers. Nurse Researcher. 2005;12: 89–91.

24. Barnes M, Cotterell P. Critical and different perspectives on user involvement. Critical perspectives on user involvement. 2012; 225–33.

25. Boote J, Baird W, Beecroft C. Public involvement at the design stage of primary health research: a narrative review of case examples. Health policy. 2010;95: 10–23.

26. Frank L, Forsythe L, Ellis L, Schrandt S, Sheridan S, Gerson J, et al. Conceptual and practical foundations of patient engagement in research at the patient-centered outcomes research institute. Quality of Life Research. 2015;24: 1033–1041.

Figure 1: Stakeholder mapping for the Nepal Pioneer Worksite Intervention study to prevent cardiometabolic risk

Click here to access/download;Figure;Figure 1.jpg

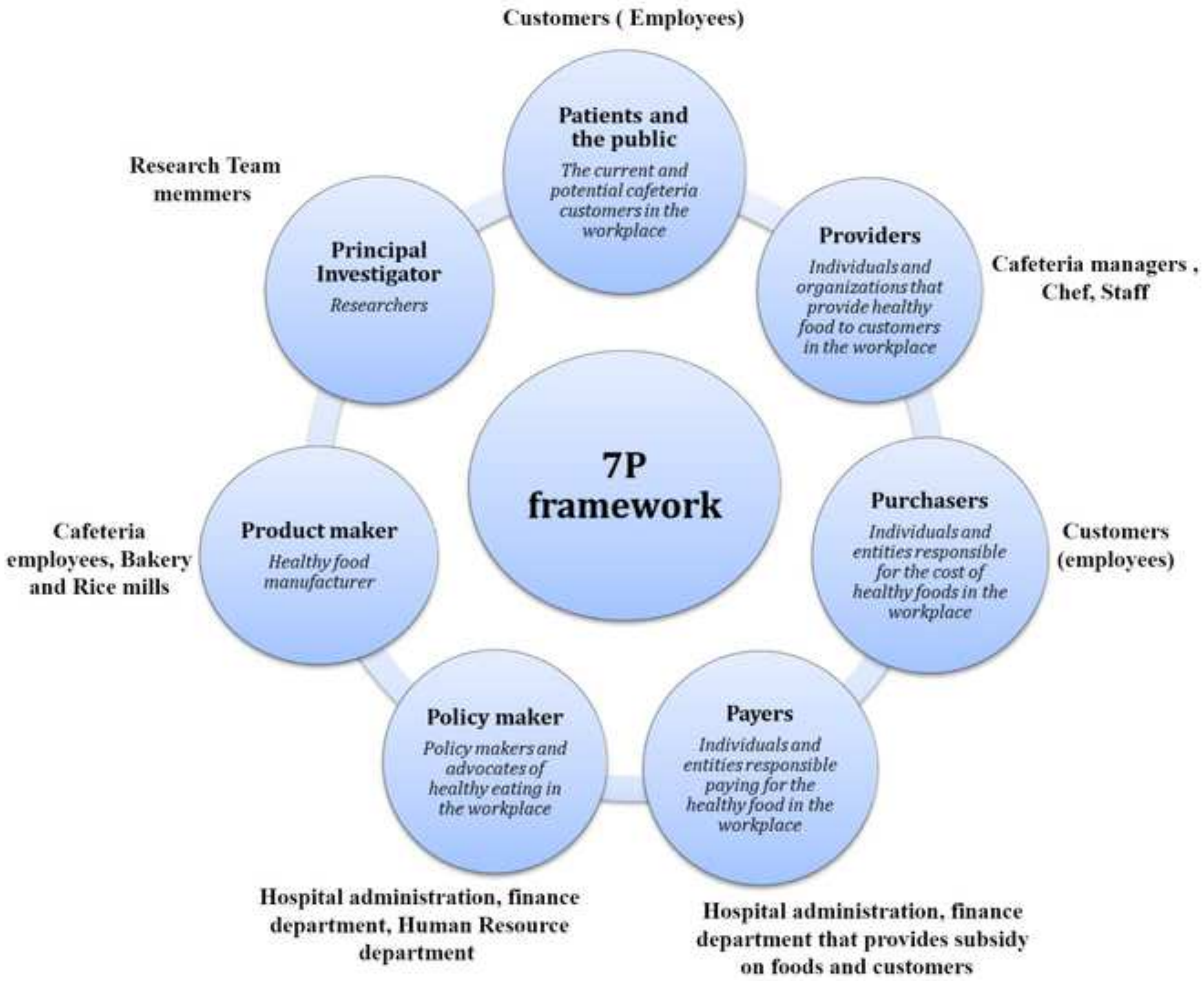

Stakeholder engagement in a hypertension and diabetes prevention research program – Description and Lessons learned

Archana Shrestha[1,2*], Dipesh Tamrakar[1], Bhawana Shrestha[1], Biraj Man Karmacharya[1], Abha Shrestha[1], Prajjwal Pyakurel[2], Donna Spiegelman[3]

~~* Corresponding author~~

~~E-mail: deararchana@gmail.com (AS)~~

[1] Department of Public Health, Kathmandu University School of Medical Sciences, Dhulikhel, Nepal

[2] Department of Community Medicine, BP Koirala Institute of Health Sciences, Dharan, Nepal

[3] Department of Biostatistics, Yale School of Public Health, New Haven, USA

* Corresponding author: Dr. Archana Shrestha; E-mail: deararchana@gmail.com

Abstract

Background

Stakeholder engagement is important from the management point of view to capture knowledge, increase ownership, reduce conflict, encourage partnership, as well as to develop an ethical perspective that facilitates inclusive decision making and promotes equity. However, there is dearth of literature in the process of stakeholder engagement. The purpose of this paper is to describe the process of increasing stakeholder engagement and highlight the lessons learnt on stakeholder engagement while designing, implementing and monitoring a study on diabetes and hypertension prevention in workplace settings in Nepal.

Methodology

We identified the stakeholders based on the 7P framework: Patients and public (clients), providers, payers, policy makers, product makers, principal investigators and purchasers. The identified stakeholders were engaged in prioritization of the research questions, planning data collection, designing, implementing and monitoring the intervention~~, interpreting the results, and results dissemination~~. Stakeholders were engaged through focus group discussions, in-depth interviews, participatory workshops, individual consultation, information sessions and representation in study team and implementation committees.

Results

The views of the stakeholders were synthesized in each step of the research process, from designing to interpreting the results. ~~The major benefits of s~~Stakeholder engagement helped to shape the methods and plan, and process for participants recruitment and data collection. In addition it enhanced adherence to intervention, ~~were increased trust in research, confidence with the researchers, enhanced~~ mutual learning, and smooth intervention adoption. The major challenges were the time-consuming nature of the process, language barriers, and the differences in health and food beliefs between researchers and stakeholders.

Conclusion

It was possible to engage and benefit from stakeholder's engagement on the design, implementation and ~~evaluation~~ monitoring of a workplace-based hypertension and diabetes management research program in Nepal.

INTRODUCTION

New scientific evidence is produced in great volume [1]~~[1]~~. However, much of this evidence has been difficult to implement in practice[2]~~ [2]~~. Stakeholder engagement can help to address this need by improving the relevance of research by increasing its transparency and accelerating the adoption into real world setting[3]~~ [3]~~. Stakeholder engagement is also important to generate knowledge, increase ownership, reduce conflict, encourage partnership, facilitate an inclusive decision making and promote equity[4].

Stakeholder engagement is promoted by health research funding organizations as well as researchers to achieve the desired goal[5]~~ [4]~~. Stakeholders can be engaged across stages of research including identifying topics, choosing hypotheses, analyzing data, and disseminating findings[6,7]~~ [5,6]~~. The levels of involvement range from consultation, to collaboration in bi-directional partnerships and ~~,~~ to leading research projects[8]~~ [7]~~.

Despite considerable attention to stakeholder engagement, there is limited information on how stakeholders are engaged. Some reviews describe the engagement in published literature and report impacts of engagement [9–12]~~ [8–11]~~. However, there is lack of reporting on how an engagement is implemented. It has been recommended that researchers systematically document and report information about engagement and its impact on individual projects[9,13]~~ [8,12]~~. Therefore, there is the need for demonstrating approaches that have been used to stakeholder engagement and opportunities to learn more about engagement in individual research projects. The purpose of this paper is to

describe the process of stakeholder engagement and the lessons learned from this process while designing, implementing and monitoring a study on diabetes and hypertension prevention in workplace settings in Nepal.

MATERIALS AND METHODS

Study setting

Nepal Pioneer Worksite Intervention Study [14][13] started in 2016 at Dhulikhel Hospital-Kathmandu University Hospital in central Nepal with a goal to prevent diabetes and hypertension among the employees of Dhulikhel Hospital. Dhulikhel Hospital is an independently owned, not-for-profit institution which was conceived and supported by the Dhulikhel community. . This study was registered on clincaltrials.gov (Identification Member:NCT03447340; Registration date: 27th February, 2018). The study was planned and executed in three phases:

Formatted: Font: (Default) Arial

Phase- 1: Formative study

We conducted a formative study to gather data useful to develop and implement an intervention to promote healthy eating at the worksite. The major aimtheme of the formative study was appropriateness to culture and geography to develop meaningful interventions at individual and organizational level. The objective was to define and assess attributes of the study participants, understand the context, tailor intervention at local context, and build relationship between researcher, study populations and other stakeholders.

Phase~~-~~2: ~~-~~Develop culturally appropriate intervention and define research attributes

In the second phase, we used the information from the formative study to develop a culturally appropriate environmental level intervention to promote healthy eating in the cafeteria of the workplace. We further used this information to define study attributes such as primary outcomes, study design, implementation and monitoring strategies, and analysis plan.

Phase~~-~~3: Test the effectiveness of the intervention

In the third phase, we ~~will~~ tested the effectiveness of the environmental level intervention using a two-step intervention study. In the first step, we ~~will~~ assessed the effectiveness of a 6-month cafeteria intervention on cardio-metabolic risk using a pre-post design. In the second step, we ~~will~~ conducted a 6-month, open-masked, two-arm randomized trial by allocating half of the participants to an individual behavioral intervention for the prevention of cardio-metabolic risk in order to estimate the additional effect of an individual level intervention. The design of the study has been published elsewhere[14] ~~[13]~~.

Stakeholder engagement

Stakeholder definition

Formatted: Font: Italic

We defined stakeholder as an individual or group affected by or who can influence the environmental intervention to promote healthy eating in the workplace.

Engagement objectives

Formatted: Font: Italic

Our objectives of stakeholder engagement were (a) to identify the specific research needs in their worksite for promoting healthy eating, (bc) to develop culturally appropriate healthy eating intervention; (c) to increase stakeholders' participation in study process such as determining study objectives and methods, describing study intervention, recruiting participants, implementing and monitoring the healthy eating intervention; (d) interpreting study results; and (e) enhancing sustainability.

Identification of stakeholders

Formatted: Font: Italic

We adopted the 7Ps framework[15] [14] that identifies key groups to consider for engagement. The first, **patients and the public**, represented the current and potential consumers of patient-centered health care and population-focused public health. The second were **providers**, including individuals and organizations that provide care to patients and populations. **Purchasers**, the individuals and entities responsible for underwriting the costs of health care, such as employers, made up the third group. The fourth group consisted of **payers** who were responsible for reimbursement of medical care, such as insurers. The fifth composed of public **policy makers** and policy advocates working in the non-governmental sector. **Product makers**, representing drug and device manufacturers, comprised the sixth group, and **principal investigators,** or other researchers, made up the seventh. Since this was not a typical patient focus study, but rather a community-based prevention trial in an institutional setting, we modified the definition of the 7Ps according to our context. (Figure 1)

[Figure 1 is here]

Formatted: Font: Bold, Italic

Figure 1: Stakeholder mapping for the Nepal Pioneer Worksite Intervention study to prevent cardiometabolic risk

Formatted: Font: Bold

Formatted: Font: Bold, Italic

Formatted: Font: 12 pt

Stakeholder engagement methods

Formatted: Font: Italic

We involved stakeholders in all three phases.

~~1.~~ **At formative study phase**

Formatted: Font: Bold

Formatted: Indent: Left: 0"

~~a.~~ ***Formation of study team:*** After mapping out the stakeholder for the study, individuals representing each stakeholder category were approached individually in-person and the study team was formed. We selected adult aged 18 years or older, employee of the hospital, who provided informed consent as stakeholder in each category. The stakeholders represented as co-investigators and advisors.

Formatted: Font: Bold, Italic

~~b.~~ ***Focus Group Discussions:*** We conducted four focus group discussions with 33 employees of the hospital to explore the current healthy eating environment, understand drivers and barriers for eating healthy, and ~~collected~~ to obtain their recommendations on improving environment to promote healthy eating at the hospital cafeteria.

Formatted: Font: Bold, Italic

The focus groups were conducted within the workplace setting in a private room to ensure confidentiality and open sharing of opinion. The FGDs were conducted in Nepali and audio taped after obtaining informed consent from the participants. The investigators, AS, or DT, moderated all FGDs in Nepali language and were

assisted by a note-taker. In each session, a brief introduction of the study and ethical considerations about maintaining confidentiality of the participants, were explained. The moderator asked open-ended questions and probed for detail information. We used iterative process by discussing each FGD immediately after completion and suggesting necessary detail probing in emerging themes from finding.

~~c.~~ ***In-depth interviews:*** We conducted 9 in-depth interviews with purposively selected hospital employees including a finance manager, a cafeteria manager, an administrative manager and 6 cafeteria operators after receiving informed consent. The investigators DT or AS conducted a semi-structured in-depth Interviews in Nepali language using a pretested interview guide in private room of the hospital. We used the iterative process for data collection. After each interview, we discussed each interview and identified the topics to be deeply explored into themes emerging in earlier interviews. All FGDs and interviews were transcribed verbatim into Nepali by trained research assistants. We used thematic analysis approach [16] ~~[15]~~ to analyze the data.

Formatted: Font: Bold, Italic

~~2.~~ **At development of research design and intervention phase**

Formatted: Font: Bold

Formatted: Indent: Left: 0"

~~a.~~ ***Workshops:*** We conducted three iterative workshops with the representation of hospital administration, cafeteria managers, cafeteria staff, nutrition department, employee and researchers. Each workshop was facilitated by DT, a co-investigator and an employee, using a workshop schedule and discussion guideline. The workshops took 3 hours. In the first hour, the facilitator explained the link between

Formatted: Font: Bold, Italic

healthy eating and cardiometabolic risks. In the second hour, the~~we~~ findings from the formative study were shared in an interactive session, and in the third hour a guided discussion with the participants was~~ere~~ conducted. The themes of the guided discussion were: components of the healthy eating interventions, mode of delivery, mode of implementation and monitoring, and method of evaluation - that led to design of the effectiveness study. The stakeholders' in workshops provided inputs in decision making. We presented the initial findings with the stakeholders and collected their feedback in the interpretation of the results.

~~b.~~ ***Food taste experiments:*** A major theme that emerged in focus group discussions, in-depth interviews and workshop was regarding acceptability of introducing whole grains in the cafeteria, especially brown rice. So, we conducted a brown rice tasting experiment with 40 employees of the hospital. Participant's perspectives on brown rice was found to be changed after tasting. Participants were willing to switch to brown rice as they found that brown rice had higher quality.[17]

c) ~~-~~ Individual consultation: The results of the formative study with further action plan were discussed individually with the hospital administrator, cafeteria manager, cafeteria chefs, nutritionist of the hospital and few employees of the hospital to get further feedback.

~~3.~~ At testing of intervention phase

Formatted: Font: Bold, Italic

Formatted: Font: Italic

Formatted: Font: Italic

Formatted: Font: Bold

Formatted: Indent: Left: 0"

~~a.~~ ***Meeting of study team:*** The study team met online every week for 30 to 60 minutes to discuss on a predetermined agenda and all decisions were taken jointly and recorded in written.

Formatted: Font: Bold, Italic

~~b.~~ ***Information sessions:*** A 20-minute information session was conducted in all departments of the hospital to provide information regarding the study and collecting feedback. About 22 sessions were conducted with participation of 4 to 20 employees in each session. In the first five minutes, the study design was explained, the next 5 minutes were dedicated for the call for participation and expectations from the participants, and 10 minutes for discussion and feedback.

Formatted: Font: Bold, Italic

~~c.~~ ***Formation of cafeteria intervention committee:*** A canteen intervention committee was formed ~~to with~~ the representation of hospital administrator, human resource department, finance department, hygiene monitoring department, non-communicable disease prevention and management department, nutrition department, canteen managers, employee and researchers. This committee steered the development, implementation and monitoring of the cafeteria intervention. The committee made the plans for the cafeteria intervention, implementation and monitoring. It meets once a month to discuss about the progress, problems and solutions.

Formatted: Font: Bold, Italic

Ethical consideration

We received approval from the institutional review board of Kathmandu University School of Medical Sciences and Nepal Health Research Council for the human subject contacts

and obtained written informed consent from the participants before collecting data for research purpose.

RESULTS

Stakeholder identification

Based on the 7P framework, the identified stakeholders are presented in ~~Table 1~~Figure 1. ~~.~~ The identified stakeholders were- customers or employees (patient and the public; purchasers); cafeteria managers, chef and staff (providers); hospital administration and finance department (payers; policy makers); Human resource department (policy maker); Cafeteria employee, bakery and Rice mills (Product maker); and Research team members (principal investigators).

~~Table 1. Stakeholder mapping for the Nepal Pioneer Worksite Intervention Study to prevention cardiometabolic risk~~

~~7P~~	~~Definition~~	~~Stakeholders in the workplace~~
~~Patients and the public~~	~~The current and potential cafeteria customers in the workplace~~	~~Customers (employees)~~
~~Providers~~	~~Individuals and organizations that provide healthy food to customers in the workplace~~	~~Cafeteria managers, chef, staff~~

Formatted: Indent: Left: 0"

Formatted Table

Formatted: Indent: Left: 0"

Formatted: Indent: Left: 0"

~~Purchasers~~	~~Individuals and entities responsible for the cost of healthy foods in the workplace~~	~~Customers (employees)~~
~~Payers~~	~~Individuals and entities responsible paying for the healthy food in the workplace~~	~~Hospital administration, finance department that provide subsidy on foods and customers~~
~~Policy makers~~	~~Policy makers and advocates of healthy eating in the workplace~~	~~Hospital administration, finance department, human resource department~~
~~Product makers~~	~~Healthy food manufacturer~~	~~Cafeteria employees, bakery, rice mills~~
~~Principal investigators~~	~~Researchers~~	~~Research Team members~~

Outcome of stakeholder engagement

Table 1 presents the stakeholder engagement in different activities within different study phases

Table 1: Stakeholder engagement in different activities within different study phases

**Study phase**	**Activity**	**Stakeholder involved**
**During Formative phase**	Formation of study team	• Principal investigator (Research team members)<br>• Purchasers (Employees including a medical doctor, a laboratory personnel, a pharmacist and a community programs expert)<br>• Payers (The Hospital administrator)<br>• Policy maker (The Hospital administrator, A nutritionist)

Formatted: Indent: Left: 0"

Formatted: Indent: Left: 0"

Formatted: Indent: Left: 0"

Formatted: Indent: Left: 0"

Formatted: Indent: Left: 0"

Formatted: Left, Line spacing: Multiple 1.15 li

Formatted: Font: Bold, Italic

Formatted: Font: 11 pt, Bold, Italic, Highlight

Formatted Table

Formatted: List Paragraph, Indent: Left: -0.08", Hanging: 0.13", Line spacing: single, Bulleted + Level: 1 + Aligned at: 0.25" + Indent at: 0.5"

Formatted: Font: 12 pt

Formatted: Font: 12 pt

Formatted: Font: 12 pt

		• Provider (A cafeteria manager)
	Focus Group Discussions	• Principal investigator (Research team members)<br>• Purchasers (Employees))
	In-depth interviews	• Provider (Cafeteria operator, Cafeteria manager)<br>• Product maker (Cafeteria Staffs, Bakery and Rice mills)<br>• Policy maker (Human resource manager)
**During development of research design and intervention phase**	Workshops	• Principal investigator (Research team members)<br>• Provider (Cafeteria operator, Cafeteria manager)<br>• Product maker (Cafeteria Staffs,<br>• Purchasers (Employees)
	Individual consultation	• Principal investigator (Research team members)<br>• Purchasers (Employees including medical doctor, laboratory personnel, pharmacist and community programs expert)<br>• Payers (Hospital administrator)<br>• Policy maker (Hospital administrator, Nutritionist)<br>• Provider (Cafeteria operator, Cafeteria manager)
**During testing of intervention phase**	Meeting of study team	• Principal investigator (Research team members)<br>• Purchasers(Employees including a medical doctor, a laboratory personnel, a pharmacist and a community programs expert)<br>• Payers (Hospital administrator)<br>• Policy maker (Hospital administrator, Nutritionist)<br>• Provider (Cafeteria operator, Cafeteria manager)
	Information sessions	• Principal investigator (Research team members)<br>• Policy makers (Departmental head and supervisors)<br>• Purchasers (Employees)
	Formation of cafeteria intervention committee:	• Product maker (Chef, Canteen operator)<br>• Policy makers (Administrative staffs, Nutritionist)<br>• Purchasers(employees including Medical doctors)

~~1.~~ During formative study phase

~~a.~~ ***Formation of study team:*** We formed a study team representing stakeholders from each category as investigator or an advisor. Investigator were those who directly participated in the day-to-day implementation of the study, compared to

Formatted: Font: 12 pt
Formatted: Font: 12 pt
Formatted: Line spacing: single
Formatted ...
Formatted: Font: 12 pt
Formatted: Font: 12 pt
Formatted: Font: 12 pt
Formatted: Font: 12 pt
Formatted: Font: 12 pt
Formatted ...
Formatted: Font: 12 pt
Formatted: Font: 12 pt
Formatted ...
Formatted: Font: 12 pt
Formatted: Not Highlight
Formatted: Font: 12 pt
Formatted: Font: 12 pt
Formatted: Font: 12 pt
Formatted: Indent: Left: -0.08", Hanging: 0.13"
Formatted ...
Formatted: Not Highlight
Formatted: Font: 12 pt
Formatted: Font: 12 pt
Formatted: Font: 12 pt
Formatted: Indent: Left: -0.08", Hanging: 0.13"
Formatted ...
Formatted: Font: 12 pt
Formatted: Font: 12 pt
Formatted: Font: 12 pt
Formatted: Font: 12 pt
Formatted: Font: 12 pt
Formatted: Font: 12 pt
Formatted: Highlight
Formatted: Indent: Left: -0.08", Line spacing: single
Formatted: Normal, Line spacing: single
Formatted: Font: Bold, Italic
Formatted: Font: Italic

advisors who provided their suggestion and guidance when asked for [18]. Four employees - a medical doctor, a laboratory personnel, a pharmacist and a community programs expert - joined the team as co-investigators. They brought their scientific expertise and unique perspective as a customer of cafeteria. The hospital administrator, a cafeteria manager and a nutritionist joined as advisor.

~~b.~~ ***Focus Group Discussions:*** Majority of participants considered that the foods that were available in the hospital cafeteria were healthy. ~~They emphasized that the foods were hygienically prepared and cheaper.~~ However, items such as confectionaries like cream donut, oily curry and meat, soda drinks, instant noodles etc. were considered unhealthy food. They emphasized that the foods were hygienically prepared and cheaper compared to hotels outside. Participants highlighted that the changes to improve food quality will be well received positively because most of them are health professionals. The most reported factors that would facilitate healthy eating were: (a) addition of healthy food options, (b) replacement of unhealthy foods with healthy options, and (c) appealing presentation of healthy foods. Active involvement of canteen management and administrator in the process of change was highly emphasized. The participants suggested to advertise healthy food options and educate both cafeteria operators and consumers on healthy eating. The major barriers to healthy eating were: (a) unavailability of healthy options, (b) lack of human resources to prepare healthy food, and (c) high price of healthy food and (d) food preferences. They suggested to add the healthy items which will demand less time and resources like adding the automated

Formatted: Font: Bold, Italic

Formatted: Font: Italic

machines such as roti makers. The support staff commented that they might not be consuming the fruits even if they are added because of the high cost. However, health professionals expressed their willingness to pay more for access to healthy food. The participants were concerned that it would be difficult to change food preferences as the consumers prefer spicy and oily foods. Some of the unhealthy foods are greatly loved such as instant noodles, samosas, cream donuts, soda drinks etc., and changing food habit is challenging.

~~c.~~ ***In-depth interviews:*** Cafeteria operator commented that the higher-level authorities should be involved in making healthy changes, deciding the menu and fixing the price. One of the canteen managers highlighted the need of a committee involving the canteen operator, administrative staff, medical doctors and nutritionist to decide on the change in menu, price, and to monitor the availability of healthy options in canteen. The canteen operators mentioned that the hospital cafeteria provides food in subsidized price and does not intend to make profit. The operators were concerned about the lack of knowledge on ~~the~~ healthy eating among the cafeteria staff and pointed that providing health education to them could~~an~~ facilitate ~~in~~ making changes. They mentioned that the cafeteria staff receive training on hygiene occasionally but have ~~not~~ never received training on healthy eating and healthy cooking. The major barrier for healthy eating reported by canteen operator were: (a) lack of adequate human resource to add food options; (b) lack of knowledge on the healthy cooking among cafeteria staff, (c) unavailability of healthy food options in the cafeteria and (d) no food supply for healthy foods such as brown rice, brown bread, organic vegetables etc. The canteen operator reported that they

Formatted: Font: Bold, Italic

Formatted: Font: Italic

are not well-staffed to provide healthy foods such as whole wheat pan bread (roti), fruits and salad as ~~they are labor intensive to prepare~~their preparation is labor intensive. ~~However, t~~The human resource manager thought that increasing efficiency of the available staff was more important than adding staff. The stakeholders contributed to the interpretation of qualitative data and contextualize the findings. The details of the FGD and Interview results are available elsewhere~~are under consideration for publication elsewhere~~ ~~.~~ [19].

~~2.~~ During development of research design and intervention phase

~~a.~~ ***Workshops:*** The discussions in the workshop were very interactive and productive. The cafeteria managers and cafeteria staffs emphasized the need for active involvement of the departments of finance ~~department~~ and administration ~~department~~ because these departments had major role deciding the menu and price of the food served in the cafeteria. This led to formation of canteen intervention committee with chairmanship of the hospital administrator and representatives from finance, human resource, nutrition, employee and researchers. There was univocal agreement in the need of adding healthy food items. However, lack of human resource in the cafeteria was a major challenge. In response, automated roti (whole wheat pan bread) maker was suggested. The details on which food ~~were~~ to add and which ~~were~~ to remove were discussed and finalized in the workshop. In addition, the ~~design~~ pre-post design was suggested in contrast to randomized trial to test the effectiveness of the changes in cafeteria on cardiometabolic risk ~~because the cafeteria~~ because it would be unethical to provide healthy food items in some

Formatted: Font: Bold, Italic

Formatted: Font: Italic

cafeterias only. Additionally, the risk of contamination was emphasized as customers do not exclusively go to one cafeteria. Consumers and chefs raised the concern on acceptability of brown rice in place of white rice. Hence, a brown rice tasting experiment was suggested.

~~b.~~ ***Individual consultation:*** Based on the findings of the FGDs, in-depth interviews and workshops, the study team prepared the description of the cafeteria intervention and proposed study design to test the effectiveness of the intervention on cardiometabolic risks. The proposal was described and discussed in-person with the stakeholders. Lack of comparison group was indicated as a weakness of the study in the initial pre-post design. As a result, six months of control period was added before intervention was implemented to be able to compare outcome measures with and without the intervention.

Formatted: Font: Bold, Italic

Formatted: Font: Italic

~~3.~~ During testing of intervention phase

~~a.~~ ***Meeting of study team:*** In the weekly meeting of the study team, the implementation strategies were discussed and finalized in minute details. This included details on disseminating information on study, call for participation, informed consent administration, data collection from the participants, enrolment planning, and intervention start dates. The internal insights of employees in the study team members guided the decision making. When the effectiveness study started, the progress on enrollment, problems and strategies were discussed weekly. For example, when there was slow blood sample collection, the representative from the laboratory suggested to provide breakfast voucher to the

Formatted: Font: Bold, Italic

Formatted: Font: Italic

participants. Adding breakfast voucher worth 70 cents in subsequent blood draw appointment speeded up the sample collection. Similarly, it was difficult to enroll the nurses ~~group~~ who had night duties. The doctor in the study team suggested ~~to collect~~ the nurse’s duty ros~~as~~ter and suggested to ~~and~~ contact them ~~them~~ immediately after their duty is over in the morning~~,~~ . This strategy~~which~~ helped to increase the participation of the nurses in the study.

~~b.~~ **Information sessions:** The information session helped to reach and interact with wider employee group. The department head provided date and venue for the information session. During the information session, feedback was obtained on the design of the study and participant recruitment. A major issue that came up was the involvement of supervisors in participants’ recruitment. The employees commented that presence of supervisors during the information session might be perceived as encouragement from them to participate. To make the participation voluntary, we conducted the sessions in absence of higher-level staff in each department, the identifiers of the participants were collected separately and were not disclosed to anyone.

~~c.~~ **Formation of cafeteria intervention committee**: The cafeteria intervention committee had key decisive role in designing, implementing, monitoring and evaluating the cafeteria intervention. Each component of the intervention was discussed and decided by the intervention committee after weighing their benefits, financial implications and perceived acceptance by the employee. The committee developed a monitoring sheet that the nutritionist and a research team member filled every week to measure the adherence to the defined intervention in all four

cafeterias. The monitoring team also interacted with the chefs, cafeteria staffs and customers informally and collected their concerns. The monitoring data were assessed and concerns were presented to the committee every month. The committee discussed the problems and strategies to address. For example, in the initial week, there was problem of supply of whole grains such as brown rice and brown bread. The committee approached the local mill and a local bakery and ordered brown rice and brown bread for the cafeteria. After adding fruits on the menu, the sales of fruits in the first two months were low. The committee decided to launch a week long campaign to promote fruits in each cafeteria which boosted their sales.

Benefits and challenges of stakeholder engagement

We are confident that our project, the project goals, methods and intervention have evolved positively based on our collaborations with stakeholders. Most importantly, their input was ~~absolutely~~ necessary in determining and describing cafeteria intervention. Their continuous involvement in monitoring helped in solving problems promptly and enhanced adherence to intervention. In addition, their input shaped methods, plans and processes for recruiting participants and data collection.

While we had a positive experience of involving stakeholder, it was not devoid of challenges. A major challenge was time availability of stakeholders as they were busy in their respective works. To address this, we scheduled meetings according to the availability of the stakeholders. We had to conduct multiple workshops and information

sessions to accommodate the time of cafeteria staff and hospital staff. This was time and resource consuming. It was also important to allow time for stakeholders to get acquainted with their new roles associated with intervention. Their knowledge of a healthy diet was dominated by the cultural norms and there was some misconception. So, we invested time and resources in training them on healthy eating. This also led to initiation of positive changes in cafeterias during the control period before the predetermined intervention date. We also felt that research priorities do not necessarily meet the priorities of the stakeholders. For example, the cafeteria staff emphasized more on the changes in physical structures such as adding culinary. There was language barrier, as researchers were comfortable using scientific jargons such as ‘epidemiology’ ‘control’ ‘confounding’, ‘subjects’ etc. making the stakeholders confused at times. It was difficult to translate or interpret these works well in local language. Additionally, the food beliefs of researchers and stakeholders were different. Stakeholders ~~had~~ strongly believe in ~~of that to~~ eating warm ‘white rice’ twice a day. In addition, ~~. Also,~~ fruits were not considered appropriate to take ~~eat at~~ during lunch. Knowledge sessions on healthy diets addressed these deep rooted ~~-~~beliefs ~~challenges~~ to some extent but changing food belief system takes longer time and more effort.

DISCUSSION

We identified employees, cafeteria managers, workplace administrators, finance manager, human resource manager, cafeteria staff and researchers as major stakeholders in the Nepal pioneer worksite intervention study to prevent cardiometabolic risks. The stakeholders were included in the study team and they ~~, who~~ brought ~~in~~ their

expertise in the subject matter and theiras well as insights as employees and customers of the cafeteria. The exploration of the facilitators and barriers to healthy eating at their worksite provided a base for defining the intervention and study attributes. Interactive workshops provided opportunity and mandate for the stakeholders to participate in decision making and strengthened the investigator-stakeholder relationship. Individual consultations helped get stronger buy-in and commitment from the higher authorities and employees. Regular meeting of the study team and canteen intervention committee guaranteed continuous engagement in problem solving and decision making. It reinforced ownership and enhanced project sustainability. The major challenges were the time-consuming nature of the process, language barriers, and differences in health and food beliefs between researchers and stakeholders.

We mapped and engaged 7 different types of stakeholders in our study. Patient-centered outcome research projects have reported engaging clinicians, caregivers, advocacy organizations and health system representatives; but only few engaged payers and policy makers[13,20] [12,16]. We incorporated the views of multiple types of stakeholders into our study during formative phase, development of intervention and study and execution of the study. For example, we formed cafeteria intervention committee based on the suggestion of cafeteria manager in in-depth interview. Brown-rice tasting experiment was also conducted to solve the concern of consumers and chefs on the acceptability of brown rice in workshop. Engagement has most frequently been reported in developing research questions[9] [8]. This study described the effort, attention, resources and flexibility for concerted engagement emphasizing on meaningful and continuous collaboration, shared leadership and decision-making power, and adaptation to the needs of stakeholders.

Other researchers have highlighted the importance of sharing power for successful research engagement[21] ~~[17]~~.

The input from different stakeholder influenced our study goals, intervention, methods and materials. This enhanced the appropriateness and relevance in the local context. Similar experience was reported by other investigators[6] ~~[5]~~. Making research more beneficiaries-centric, culturally relevant and accessible to potential research participants have been reported to refine proposals, recruitment materials, and participant compensation[22] ~~[18]~~. In our project, the stakeholders contributed to the interpretation of qualitative data and contextualize the findings, which was also reported by other researchers[11] ~~[10]~~. This is consistent with the available evidence on positive impacts of engaged research[6,12] ~~[5,11].~~ .

The stakeholder engagement has contributed positively in our study from formative phase to execution. Various levels of stakeholder involvement, such as consultation, collaboration or user-control has been proposed[23] ~~[20]~~. However, caution has been recommended against assuming that involving higher levels is better[24] ~~[21]~~. Some researchers in a national survey from UK claimed that decisions about the appropriateness of consumer involvement in research should be made on a case-by-case basis [13] ~~[12]~~.

The major challenges that we faced were the time-consuming nature of the process, language barriers, and differences in health and food beliefs between researchers and stakeholders. Similar challenges have been reported in other studies[6,25] ~~[5,22].~~ Lack of time—both for stakeholders and researchers—was the most frequently noted challenge and was identified as a top barrier to engagement in a recent survey of

investigators[25] ~~[22]~~. Thus, it is crucial to develop strategies that maximize stakeholder input with minimum time and resources[26] ~~[23]~~.

The generalizability of our findings is limited because of the unique setting where the research was conducted. Because it was a university hospital, we had an advantage of including the employees with specific research expertise as co-investigators. The stakeholders had more interest in and experience with the health interventions and research. Thus, their knowledge and attitude about engagement may be more positive.

CONCLUSION

It was possible to engage and benefit from stakeholder's engagement on the design, implementation and ~~evaluation~~ monitoring of a workplace-based hypertension and diabetes management research program in Nepal. We recommend ongoing program development and implementation by stakeholder input. In future, we plan to evaluate the influence of stakeholders on the research project and assess the engagement and effectiveness through their perspective, as well as longer term outcomes on health decision making beyond the conduction of research.

ACKNOWLEDGMENTS

We would like to acknowledge the Dhulikhel Hospital – Kathmandu University Hospital for their permission and cooperation to conduct this study. We appreciate the contribution of all the study participants.

AUTHOR CONTRIBUTIONS

Conceptualization: Archana Shrestha, Dipesh Tamrakar, Donna Spiegelman

Formal analysis: Bhawana Shrestha, Biraj Man Karmacharya, Abha Shrestha, Prajjwal Pyakurel

Writing – original draft: Archana Shrestha

Writing – review & editing: Archana Shrestha, Dipesh Tamrakar, Bhawana Shrestha, Biraj Man Karmacharya, Abha Shrestha, Prajjwal Pyakurel, Donna Spiegelman

REFERENCES

1. Joyce MC. How will I ever keep up with pharmacy changes? Pharmacy Today. 2015;21(1):1.

2. Zilberberg MD. The clinical research enterprise: time to change course? JAMA. 2011 Feb 9;305(6):604–5.

3. Ray KN, Miller E. Strengthening stakeholder-engaged research and research on stakeholder engagement. J Comp Eff Res. 2017 Jun;6(4):375–89.

4. Kok MO, Gyapong JO, Wolffers I, Ofori-Adjei D, Ruitenberg J. Which health research gets used and why? An empirical analysis of 30 cases. Health Res Policy Syst [Internet]. 2016;14(1). Available from: http://dx.doi.org/10.1186/s12961-016-0107-2

5. Shippee ND, Domecq Garces JP, Prutsky Lopez GJ, Wang Z, Elraiyah TA, Nabhan M, et al. Patient and service user engagement in research: a systematic review and synthesized framework. Health Expect. 2015 Oct;18(5):1151–66.

6. Mullins CD, Abdulhalim AM, Lavallee DC. Continuous patient engagement in comparative effectiveness research. JAMA. 2012 Apr 18;307(15):1587–8.

7. Lowes L, Hulatt I. Involving Service Users in Health and Social Care Research. Routledge; 2013. 214 p.

8. Concannon TW, Fuster M, Saunders T, Patel K, Wong JB, Leslie LK, et al. A

Formatted: Indent: Left: 0", First line: 0"

systematic review of stakeholder engagement in comparative effectiveness and patient-centered outcomes research. J Gen Intern Med. 2014 Dec;29(12):1692–701.

9. Forsythe LP, Szydlowski V, Murad MH, Ip S, Wang Z, Elraiyah TA, et al. A systematic review of approaches for engaging patients for research on rare diseases. J Gen Intern Med. 2014 Aug;29 Suppl 3:S788–800.

10. Boote J, Baird W, Sutton A. Involving the public in systematic reviews: a narrative review of organizational approaches and eight case examples. J Comp Eff Res. 2012 Sep;1(5):409–20.

11. Brett J, Staniszewska S, Mockford C, Herron-Marx S, Hughes J, Tysall C, et al. Mapping the impact of patient and public involvement on health and social care research: a systematic review. Health Expect. 2014 Oct;17(5):637–50.

12. Barber R, Boote JD, Cooper CL. Involving consumers successfully in NHS research: a national survey. Health Expect. 2007 Dec;10(4):380–91.

13. Shrestha A, Tamrakar D, Karmacharya BM, Shrestha A, Shrestha R, Bhatta RD, et al. Nepal Pioneer Worksite Intervention Study to lower cardio-metabolic risk factors: design and protocol. BMC Cardiovasc Disord. 2019 Feb 28;19(1):48.

14. Concannon TW, Meissner P, Grunbaum JA, McElwee N, Guise J-M, Santa J, et al. A new taxonomy for stakeholder engagement in patient-centered outcomes research. J Gen Intern Med. 2012 Aug;27(8):985–91.

15. Boyatzis RE. Transforming Qualitative Information: Thematic Analysis and Code Development. SAGE; 1998. 184 p.

16. Forsythe LP, Ellis LE, Edmundson L, Sabharwal R, Rein A, Konopka K, et al. Patient and Stakeholder Engagement in the PCORI Pilot Projects: Description and Lessons Learned. J Gen Intern Med. 2016 Jan;31(1):13–21.

17. Westfall JM, Fagnan LJ, Handley M, Salsberg J, McGinnis P, Zittleman LK, et al. Practice-based research is community engagement. J Am Board Fam Med. 2009 Jul;22(4):423–7.

18. Joosten YA, Israel TL, Williams NA, Boone LR, Schlundt DG, Mouton CP, et al. Community Engagement Studios: A Structured Approach to Obtaining Meaningful Input From Stakeholders to Inform Research. Acad Med. 2015 Dec;90(12):1646–50.

19. Exploring Impact: Public Involvement in NHS, Public Health and Social Care Research. 2009.

20. Newell C. Involving the Public in NHS, Public Health, and Social Care Research: Briefing Notes for Researchers (2nd edition). Health Expect. 2005;8(1):91–2.

21. Barnes M, Cotterell P. Critical Perspectives on User Involvement. Policy Press; 2011. 269 p.

22. Boote J, Baird W, Beecroft C. Public involvement at the design stage of primary health research: A narrative review of case examples. Health Policy. 2010;95(1):10–23.

~~23. Frank L, Forsythe L, Ellis L, Schrandt S, Sheridan S, Gerson J, et al. Conceptual and practical foundations of patient engagement in research at the patient-centered outcomes research institute. Qual Life Res. 2015 May;24(5):1033–41.~~

Formatted: Heading 1, Line spacing: Double

1. Joyce MC. How will I ever keep up with pharmacy changes? Pharmacy Today. 2015;21: 1.
2. Zilberberg MD. The clinical research enterprise: time to change course? Jama. 2011;305: 604–605.
3. Ray KN, Miller E. Strengthening stakeholder-engaged research and research on stakeholder engagement. Journal of comparative effectiveness research. 2017;6: 375–389.
4. Mathur VN, Price ADF, Austin S. Conceptualizing stakeholder engagement in the context of sustainability and its assessment. Construction Management and Economics. 2008;26: 601–609. doi:10.1080/01446190802061233
5. Kok MO, Gyapong JO, Wolffers I, Ofori-Adjei D, Ruitenberg J. Which health research gets used and why? An empirical analysis of 30 cases. Health research policy and systems. 2016;14: 1–18.
6. Shippee ND, Domecq Garces JP, Prutsky Lopez GJ, Wang Z, Elraiyah TA, Nabhan M, et al. Patient and service user engagement in research: a systematic review and synthesized framework. Health Expectations. 2015;18: 1151–1166.
7. Mullins CD, Abdulhalim AM, Lavallee DC. Continuous patient engagement in comparative effectiveness research. Jama. 2012;307: 1587–1588.
8. Lowes L, Hulatt I. Involving service users in health and social care research. Routledge; 2013.
9. Concannon TW, Fuster M, Saunders T, Patel K, Wong JB, Leslie LK, et al. A systematic review of stakeholder engagement in comparative effectiveness and patient-centered outcomes research. Journal of general internal medicine. 2014;29: 1692–1701.
10. Forsythe LP, Szydlowski V, Murad MH, Ip S, Wang Z, Elraiyah TA, et al. A systematic review of approaches for engaging patients for research on rare diseases. Journal of general internal medicine. 2014;29: 788–800.

11. Boote J, Baird W, Sutton A. Involving the public in systematic reviews: a narrative review of organizational approaches and eight case examples. Journal of comparative effectiveness research. 2012;1: 409–420.

12. Brett J, Staniszewska S, Mockford C, Herron-Marx S, Hughes J, Tysall C, et al. Mapping the impact of patient and public involvement on health and social care research: a systematic review. Health expectations. 2014;17: 637–650.

13. Barber R, Boote JD, Cooper CL. Involving consumers successfully in NHS research: a national survey. Health Expectations. 2007;10: 380–391.

14. Shrestha A, Tamrakar D, Karmacharya BM, Shrestha A, Shrestha R, Bhatta RD, et al. Nepal Pioneer Worksite Intervention Study to lower cardio-metabolic risk factors: design and protocol. BMC cardiovascular disorders. 2019;19: 1–10.

15. Concannon TW, Meissner P, Grunbaum JA, McElwee N, Guise J-M, Santa J, et al. A new taxonomy for stakeholder engagement in patient-centered outcomes research. Journal of general internal medicine. 2012;27: 985–991.

16. Boyatzis RE. Transforming qualitative information: Thematic analysis and code development. sage; 1998.

17. Gyawali P, Tamrakar D, Shrestha A, Shrestha H, Karmacharya S, Bhattarai S, et al. Consumer acceptance and preference for brown rice—A mixed-method qualitative study from Nepal. Food Science & Nutrition. 2022; fsn3.2803. doi:10.1002/fsn3.2803

18. Sciences NA of, Science NA of E (US) and I of M (US) C on, Engineering, Policy and P. ADVISING AND MENTORING. On Being a Scientist: A Guide to Responsible Conduct in Research: Third Edition. National Academies Press (US); 2009. Available: https://www.ncbi.nlm.nih.gov/books/NBK214555/

19. Tamrakar D, Shrestha A, Rai A, Karmacharya BM, Malik V, Mattei J, et al. Drivers of healthy eating in a workplace in Nepal: a qualitative study. BMJ Open. 2020;10: e031404. doi:10.1136/bmjopen-2019-031404

20. Forsythe LP, Ellis LE, Edmundson L, Sabharwal R, Rein A, Konopka K, et al. Patient and stakeholder engagement in the PCORI pilot projects: description and lessons learned. Journal of general internal medicine. 2016;31: 13–21.

21. Westfall JM, Fagnan LJ, Handley M, Salsberg J, McGinnis P, Zittleman LK, et al. Practice-based research is community engagement. The Journal of the American Board of Family Medicine. 2009;22: 423–427.

22. Joosten YA, Israel TL, Williams NA, Boone LR, Schlundt DG, Mouton CP, et al. Community engagement studios: a structured approach to obtaining meaningful input from stakeholders to inform research. Academic Medicine. 2015;90: 1646.

23. Dent H. Involving the Public in NHS, Public Health and Social Care Research: Briefing notes for researchers. Nurse Researcher. 2005;12: 89–91.

24. Barnes M, Cotterell P. Critical and different perspectives on user involvement. Critical perspectives on user involvement. 2012; 225–33.

25. Boote J, Baird W, Beecroft C. Public involvement at the design stage of primary health research: a narrative review of case examples. Health policy. 2010;95: 10–23.

26. Frank L, Forsythe L, Ellis L, Schrandt S, Sheridan S, Gerson J, et al. Conceptual and practical foundations of patient engagement in research at the patient-centered outcomes research institute. Quality of Life Research. 2015;24: 1033–1041.

Legends

Figure 1: Stakeholder mapping for the Nepal Pioneer Worksite Intervention study to prevent cardiometabolic risk

Formatted: Font: Bold

Formatted: Font: Italic

Response to Reviewers

11/05/2022

To

Editors and team

PLOS ONE journal

Subject: Re-submission of an original research article to PLOS ONE journal addressing the reviewer's comment

Dear sir/madam,

It is our pleasure to re-submit to you and the editorial teams our manuscript entitled "**Stakeholder Engagement in a Hypertension and Diabetes Prevention Research Program – Description and Lessons Learned**". The paper aims to describe the stakeholder engagement process and the lessons learned from this process while designing, implementing and monitoring a study on diabetes and hypertension prevention in workplace settings in Nepal. It provides important insights for future researchers that aim to engage stakeholders in national-level preventive programs in health.

We would like to thank all the reviewers for their constructive comments. The responses to the reviewer's comments are addressed individually below:

Reviewer 1

This manuscript describes stakeholder engagement activities and lessons learned in diabetes and hypertension prevention study in workplace settings in Nepal. The authors describe stakeholder engagement activities during the formative, intervention development, and study conduct phases of this project. In general, this paper could benefit from a grammatical review as there are several errors throughout.

Additionally, the literature review should be updated to reflect current gaps, as there are several existing articles to date that offer engagement implementation examples, which was stated as a gap and strength of current paper. The impact of stakeholder engagement on research activities and translation is an area for further contribution and an opportunity where this manuscript could offer insight. Areas of stakeholder impact should be more clearly identified in the paper. In addition to above, consider the following to strengthen this manuscript:

-Consider re-designing Table 1 into a Figure and move to first presentation of stakeholders using 7P Framework in Methods section (not in Results section as current placement).

>> The Table 1 is converted to figure and shifted to methods section

-Although it was stated that results from qualitative work is under consideration for publication elsewhere, this section could benefit from including more specifics regarding questions asked during focus group sessions/in-depth interviews with stakeholders and/or qualitative supporting quotes from discussions, since these informed intervention. Who came up with interview questions? How did the research team decide which feedback to implement?

>> T***he result and methods are available in detailed in the paper below and it is now cited.***

> ***Tamrakar D, Shrestha A, Rai A, Karmacharya BM, Malik V, Mattei J, et al. Drivers of healthy eating in a workplace in Nepal: a qualitative study. BMJ Open. 2020;10: e031404. doi:10.1136/bmjopen-2019-031404***

-Consider creating a new Table 1 in Results that includes stakeholder type and the actual activities that the stakeholders were engaged in (i.e. formative and then subheading for which activity i.e. focus group). It was not clear within text the myriad of stakeholders who participated in the various study processes. This table could also include a column "Impact" to highlight how stakeholder contributions informed the study and in what way(s).

>>Table is generated as per your suggestions

Reviewer 2

An important work in the area stakeholder engagement. Generally, well written but needs some language editing and improvement in flow in some sections.

Abstract

Lines 39-41. What is described does not include interpreting the results and results dissemination.

>> *It is revised as per your suggestion*

Lines 47: I am not sure increased trust in research and confidence with the researchers is a result or what can be inferred (or assumed) given the processes and outcomes. What evidence do you have for this allusion?

>> *It is revised as per your suggestion*

Line 54: The manuscript has not reported on engaging the stakeholders in the evaluation may be monitoring.

>>*The comment is addressed*

Introduction

Line 62: Give reference

>> *Reference is added*

See comment on line 67

>> *comment addressed*

Materials and methods

-Line 88: Aim would be more appropriate than “theme”

>> *“theme” is changed to “aim”*

-Sub-titles e.g., Phase 1 Formative study would be better be presented as Phase 1: Formative study

-Lines 106 to lines 202: Flow in stakeholder engagement needs to be improved for easier reading. I suggest a main sub-title “Stakeholder engagement” and smaller sub-titles as follows: stakeholder definition, engagement objectives, stakeholder identification, and stakeholder methods of engagement.

>> *Its flow is changed according to the suggestion provided.*

-Not sure it is necessary to number the phases e.g., line 134

>>The phrases are now un-numbered

-Lines 135-137. What criteria did you use to identify/include the individuals from each stakeholder category after conducting the mapping?. This also applies to those included in focus groups discussion (FDGs).

>> Added to the manuscript. We selected adults aged 18 years or older, employee of the hospital, who provided informed consent in each stakeholder category.

-Purposive selection for interviews- what specific information did you expect that these participants could be having unlike others

>> ***They had unique experience of making first hand decisions and implementation at the cafeteria and other wellness programs for the employees. These interviewees brought their perspectives on the decision making system, how it affected healthy food choices in the cafeteria and other administrative decisions regarding employee's health. This would provide insights into the facilitators and barriers to healthy eating in the workplace environment.***

-I think its important to mention qualitative methods were used for FDGs and the in-depth interviews- as the methods hold specific benefits and give support from the literature on the choice of method

>> >> T***he result and methods for FGDs and in depth interviews are available in detailed in the paper below and it is now cited.***

> ***Tamrakar D, Shrestha A, Rai A, Karmacharya BM, Malik V, Mattei J, et al. Drivers of healthy eating in a workplace in Nepal: a qualitative study. BMJ Open. 2020;10: e031404. doi:10.1136/bmjopen-2019-031404***

-Line 172: I think topic rather than theme will be more appropriate

>> The word theme is replaced by the topic

-What was the stakeholders' role in the workshops?

*>>***The stakeholders provided their inputs in decision making. We presented the initial findings with the stakeholders and collected their feedback in the interpretation of the results.***

Results

-Line 211: What informed/guided the inclusion to a certain category (investigator vs. advisor). Any evidence from literature?

>> Investigator were those who directly participated in the day-to-day implementation of the study, compared to advisors who provided their suggestions and guidances when asked for.

-As a reader, I was expecting more details of the FDGs and interviews, but I saw at the end the statement that the results are under consideration for publication elsewhere. Just wondering, the implication of this given that data needs to be availed by authors.

>>>> ***The result and methods are available in detailed in the paper below and it is now cited.***

> ***Tamrakar D, Shrestha A, Rai A, Karmacharya BM, Malik V, Mattei J, et al. Drivers of healthy eating in a workplace in Nepal: a qualitative study. BMJ Open. 2020;10: e031404. doi:10.1136/bmjopen-2019-031404***

-Lines 231-232: They suggested to add the healthy items which will demand less time and resources like adding the automated machines such as roti makers. Rephrase I don't think automated machines needs less resource (may be human but not financial)

>> ***The sentence is rephrased as "They suggested to add the healthy items which will demand less time and human resources like adding the automated machines such as roti makers."***

-Interchange order of sentences on lines 218-219 and lines 219-221 for better flow.

>> The sentences are interchanged

-What was the outcome of the food testing and how did it inform the intervention?

>> ***Added to the manuscript. Participant's perspectives on brown rice was found to be changed after tasting. Participants were willing to switch to brown rice as they found that brown rice had higher quality.[17]***

-Rephrase lines 356-358 for clarity

>>The line is rephrased for clarity

-Lines 392-394: There is nowhere that is reported how the stakeholders were involved in interpreting the data.

>> The comment is addressed in the manuscript.

Discussion

Lines 379 to 387: It would be useful if the authors could highlight on how they incorporated the views of the stakeholders in each step, probably in a Table format. For example; What was the final goal and what stakeholder input lead to that outcome.

>> We added some ways that we used to incorporate views of stakeholders. (. For example, we formed cafeteria intervention committee based on the suggestion of cafeteria manager in in-depth interview. Brown-rice tasting experiment was also conducted to solve the concern of consumers and chefs on the acceptability of brown rice in workshop.)

Other comments

1. **Please ensure you have included the registration number for the clinical trial referenced in the manuscript.**

   The registration number is added in the manuscript. This study was registered on clincaltrials.gov (Identification Member:NCT03447340; Registration date: 27th February, 2018).

2. **To help ensure that the wording of your manuscript is suitable for publication, would you please also add this statement at the beginning of the Methods section of your manuscript file.**

   The ethical statement is added in the publication.

   Ethical consideration: We received approval from the institutional review board of Kathmandu University School of Medical Sciences and Nepal Health Research Council for the human subject contacts and obtained written informed consent from the participants before collecting data for research purpose.

3. Data availability statement.
   The dataset is uploaded is OSF. The link to dataset is https://osf.io/bqsrd/

I think it is important for the reader to know where the project is at the moment- is still being implemented and prospect of sustainability given that its one of the objective of involving stakeholders

We look forward to your comments and remain with best regards;

Yours sincerely,

Dr. Archana Shrestha

Department of Public Health

Kathmandu University School of Medical Sciences

Dhulikhel, Nepal
