# Supplementary material for: Stakeholder engagement in a hypertension and diabetes prevention research program: Description and lessons learned
Source: PLoS One. 2022 Oct 20;17(10):e0276478. doi: 10.1371/journal.pone.0276478 (PMC9584412; doi:10.1371/journal.pone.0276478)
Supplement: Attachment — Submitted filename: PONE-D-21-05456_R2.pdf-JM-06.pdf [file pone.0276478.s006.pdf]

PLOS ONE

Stakeholder engagement in a hypertension and diabetes prevention research program

--Manuscript Draft--

**Manuscript Number:**	PONE-D-21-05456R2
**Article Type:**	Research Article
**Full Title:**	Stakeholder engagement in a hypertension and diabetes prevention research program
**Short Title:**	Stakeholder engagement
**Corresponding Author:**	Archana Shrestha<br>Kathmandu University Hospital: Dhulikhel Hospital<br>Dhulikhel, Bagmati NEPAL
**Keywords:**	stakeholder engagement; Hypertension; Diabetes; Workplace settings; Nepal
**Abstract:**	Background<br>Stakeholder engagement is important from the management point of view to capture knowledge, increase ownership, reduce conflict, encourage partnership, as well as to develop an ethical perspective that facilitates inclusive decision making and promotes equity. However, there is dearth of literature in the process of stakeholder engagement. The purpose of this paper is to describe the process of increasing stakeholder engagement and highlight the lessons learnt on stakeholder engagement while designing, implementing and monitoring a study on diabetes and hypertension prevention in workplace settings in Nepal.<br>Methodology<br>We identified the stakeholders based on the 7P framework: Patients and public (clients), providers, payers, policy makers, product makers, principal investigators and purchasers. The identified stakeholders were engaged in prioritization of the research questions, planning data collection, designing, implementing and monitoring the intervention. Stakeholders were engaged through focus group discussions, in-depth interviews, participatory workshops, individual consultation, information sessions and representation in study team and implementation committees.<br>Results<br>The views of the stakeholders were synthesized in each step of the research process, from designing to interpreting the results. Stakeholder engagement helped to shape the methods and plan, and process for participant's recruitment and data collection. In addition, it enhanced adherence to intervention, mutual learning, and smooth intervention adoption. The major challenges were the time-consuming nature of the process, language barriers, and the differences in health and food beliefs between researchers and stakeholders.<br>Conclusion<br>It was possible to engage and benefit from stakeholder's engagement on the design, implementation and monitoring of a workplace-based hypertension and diabetes management research program in Nepal.
**Order of Authors:**	Archana Shrestha
	Dipesh Tamrakar
	Bhawana Shrestha
	Biraj Man Karmacharya
	Abha Shrestha
	Prajjwal Pyakurel
	Donna Spiegelman
**Response to Reviewers:**	Response to Reviewers<br><br>08/22/2022<br>To<br>Editors and team<br>PLOS ONE journal

Powered by Editorial Manager® and ProduXion Manager® from Aries Systems Corporation

Subject: Re-submission of an original research article to PLOS ONE journal addressing the reviewer's comment

Dear sir/madam,
It is our pleasure to re-submit our manuscript entitled “Stakeholder Engagement in a Hypertension and Diabetes Prevention Research Program” to the editorial teams. The paper aims to describe the stakeholder engagement process and the lessons learned from this process while designing, implementing and monitoring a study on diabetes and hypertension prevention in workplace settings in Nepal. It provides important insights for future researchers that aim to engage stakeholders in national-level preventive programs in health.
We would like to thank all the reviewers for their constructive comments. The responses to the reviewer’s comments are addressed individually below:
Reviewer 2
General:
An important topic that will contribute to stakeholder engagement in interventions knowledge base. But unless, I have not understood the title “It is description & lessons learnt” As a reader I don’t think I still can clearly pinpoint the lessons learnt unless in the manuscript it is equivalent to benefits & challenges. Wonder if the title should not be Description & experiences?
>> Based on your suggestions we removed Description & experiences from title.
Generally, well written. However, the manuscript can benefit from some editing e.g. Line 143: word interview should not be started with upper case. Line 144: Add “a” between guide and in private room. More information on specific places to correct are given in comments on the manuscript itself.
>>Edited based on your comments
I see some of the work mentioned in the manuscript has been published, for example the FDGs & in-depth interviews. I think it is important that the reader understands from the word go, that details are reported somewhere. For example, I had a question on the number of participants per FDG, and wondering about the participant’s quotes, but I see these details are in reference 19.
>> We have added brief of the methods and results
I think what would important is to highlight what informed the authors decisions- for example to form a study committee, use focus groups/workshops and not any methods for engagement, in case a reader would want to conduct a similar stakeholder engagement but still briefly report how these activities were done. I believe you were guided by literature.
>> It is mentioned in under Engagement objectives heading.
2 Introduction: The statement “New scientific evidence” appears incomplete without saying what kind of evidence. For example, is it health promotion/medical intervention effectiveness?
>> Addressed

Methods:
Lines 122- 126: The formation of the study team is unclear. I would believe some stakeholder categories had more than one person. How did you decide which representative(s) to approach? Are there people who declined to take part, since you have said those who provided informed consent? The last part “The stakeholders represented as co-investigators and advisors” need to be rephrased for clarity. [Probably referring to your published work could help as well]. I think its important to mention the rationale for the constituents of study team in the methods section
>> The formation of the study team is now rephrased and edited to make more clear statement
Workshops: As a reader I will be interested to know the aim of the workshop. It may be good to give the ethics approval number The authors should detail how stakeholders were involved in the interpretation of the results.
>>Aim of workshop and approval number are added in the manuscript
Line 352- put a reference as this is documented in the literature. I believe the first part of the discussion i.e. 356 to 367, there would be literature documenting these strategies in other studies. If not the authors should indicate there is no other studies identified that have used the same strategies as the current study.
>>Reference is added to the line 352.

Powered by Editorial Manager® and ProduXion Manager® from Aries Systems Corporation

	Conclusion does not include any comment on lessons learnt.<br>>> We removed lesson learnt from title. And hence lesson learnt is not placed in the conclusion section.<br><br>We look forward to your comments and remain with best regards;<br><br>Yours sincerely,<br>Dr. Archana Shrestha<br>Department of Public Health<br>Kathmandu University School of Medical Sciences<br>Dhulikhel, Nepal
**Additional Information:**	
**Question**	**Response**
**Financial Disclosure**<br><br>Enter a financial disclosure statement that describes the sources of funding for the work included in this submission. Review the submission guidelines for detailed requirements. View published research articles from *PLOS ONE* for specific examples.<br><br>This statement is required for submission and **will appear in the published article** if the submission is accepted. Please make sure it is accurate.<br><br>**Unfunded studies**<br>Enter: *The author(s) received no specific funding for this work.*<br><br>**Funded studies**<br>Enter a statement with the following details:<br>• Initials of the authors who received each award<br>• Grant numbers awarded to each author<br>• The full name of each funder<br>• URL of each funder website<br>• Did the sponsors or funders play any role in the study design, data collection and analysis, decision to publish, or preparation of the manuscript?<br>• **NO** - Include this sentence at the end of your statement: *The funders had no role in study design, data collection and analysis, decision to publish, or preparation of the manuscript.*<br>• **YES** - Specify the role(s) played.<br><br>* typeset	•DS, AS, DT<br>•Award no: 5DP1ES02545903<br>•National Institute of Health<br>•https://www.nih.gov/about-nih<br>•The funders had no role in study design, data collection and analysis, decision to publish, or preparation of the manuscript.
**Competing Interests**	The authors have declared that no competing interests exist.

Use the instructions below to enter a competing interest statement for this submission. On behalf of all authors, disclose any competing interests that could be perceived to bias this work—acknowledging all financial support and any other relevant financial or non-financial competing interests.<br><br>This statement is required for submission and **will appear in the published article** if the submission is accepted. Please make sure it is accurate and that any funding sources listed in your Funding Information later in the submission form are also declared in your Financial Disclosure statement.<br><br>View published research articles from *PLOS ONE* for specific examples.<br><br>**NO authors have competing interests**<br><br>Enter: *The authors have declared that no competing interests exist.*<br><br>**Authors with competing interests**<br><br>Enter competing interest details beginning with this statement:<br><br>*I have read the journal's policy and the authors of this manuscript have the following competing interests: [insert competing interests here]*<br><br>* typeset	
**Ethics Statement**<br><br>Enter an ethics statement for this submission. This statement is required if the study involved:<br><br>• Human participants<br>• Human specimens or tissue<br>• Vertebrate animals or cephalopods<br>• Vertebrate embryos or tissues	We received approval from the institutional review board of Kathmandu University School of Medical Sciences and Nepal Health Research Council for the human subject contacts and obtained written informed consent from the participants before collecting data for research purpose.

Powered by Editorial Manager® and ProduXion Manager® from Aries Systems Corporation

• Field research<br><br>Write "N/A" if the submission does not require an ethics statement.<br><br>General guidance is provided below. Consult the submission guidelines for detailed instructions. **Make sure that all information entered here is included in the Methods section of the manuscript.**	

Powered by Editorial Manager® and ProduXion Manager® from Aries Systems Corporation

**Format for specific study types**<br><br>**Human Subject Research (involving human participants and/or tissue)**<br>• Give the name of the institutional review board or ethics committee that approved the study<br>• Include the approval number and/or a statement indicating approval of this research<br>• Indicate the form of consent obtained (written/oral) or the reason that consent was not obtained (e.g. the data were analyzed anonymously)<br><br>**Animal Research (involving vertebrate animals, embryos or tissues)**<br>• Provide the name of the Institutional Animal Care and Use Committee (IACUC) or other relevant ethics board that reviewed the study protocol, and indicate whether they approved this research or granted a formal waiver of ethical approval<br>• Include an approval number if one was obtained<br>• If the study involved *non-human primates*, add *additional details* about animal welfare and steps taken to ameliorate suffering<br>• If anesthesia, euthanasia, or any kind of animal sacrifice is part of the study, include briefly which substances and/or methods were applied<br><br>**Field Research**<br><br>Include the following details if this study involves the collection of plant, animal, or other materials from a natural setting:<br>• Field permit number<br>• Name of the institution or relevant body that granted permission	
**Data Availability**<br><br>Authors are required to make all data underlying the findings described fully available, without restriction, and from the time of publication. PLOS allows rare exceptions to address legal and ethical concerns. See the PLOS Data Policy and FAQ for detailed information.	Yes - all data are fully available without restriction

A Data Availability Statement describing where the data can be found is required at submission. Your answers to this question constitute the Data Availability Statement and **will be published in the article**, if accepted.<br><br>**Important:** Stating 'data available on request from the author' is not sufficient. If your data are only available upon request, select 'No' for the first question and explain your exceptional situation in the text box.<br><br>Do the authors confirm that all data underlying the findings described in their manuscript are fully available without restriction?	
**Describe where the data may be found in full sentences. If you are copying our sample text, replace any instances of XXX with the appropriate details.**<br><br>• If the data are **held or will be held in a public repository**, include URLs, accession numbers or DOIs. If this information will only be available after acceptance, indicate this by ticking the box below. For example: *All XXX files are available from the XXX database (accession number(s) XXX, XXX.*).<br>• If the data are all contained **within the manuscript and/or Supporting Information files**, enter the following: *All relevant data are within the manuscript and its Supporting Information files.*<br>• If neither of these applies but you are able to provide **details of access elsewhere**, with or without limitations, please do so. For example:<br><br>*Data cannot be shared publicly because of [XXX]. Data are available from the XXX Institutional Data Access / Ethics Committee (contact via XXX) for researchers who meet the criteria for access to confidential data.*<br><br>*The data underlying the results presented in the study are available from (include the name of the third party*	**PA at Accept: Please ensure data are publicly available**<br><br>The data has been deposited to Open Science Framework (OSF) public repository with URL: https://osf.io/bqsrd/

and contact information or URL).<br>• This text is appropriate if the data are owned by a third party and authors do not have permission to share the data.<br><br>* typeset	
Additional data availability information:	

Powered by Editorial Manager® and ProduXion Manager® from Aries Systems Corporation

Manuscript
Click here to access/download;Manuscript;2 Stakeholder Engagement_Manuscript.docx

Stakeholder engagement in a hypertension and diabetes prevention research program

Archana Shrestha[1,2*], Dipesh Tamrakar[1], Bhawana Shrestha[1], Biraj Man Karmacharya[1], Abha Shrestha[1], Prajjwal Pyakurel[2], Donna Spiegelman[3]

[1] Department of Public Health, Kathmandu University School of Medical Sciences, Dhulikhel, Nepal

[2] Department of Community Medicine, BP Koirala Institute of Health Sciences, Dharan, Nepal

[3] Department of Biostatistics, Yale School of Public Health, New Haven, USA

* Corresponding author: Dr. Archana Shrestha; E-mail: dearatchana@gmail.com

Abstract

Background

Stakeholder engagement is important from the management point of view to capture knowledge, increase ownership, reduce conflict, encourage partnership, as well as to develop an ethical perspective that facilitates inclusive decision making and promotes equity. However, there is dearth of literature in the process of stakeholder engagement. The purpose of this paper is to describe the process of increasing stakeholder engagement and highlight the lessons learnt on stakeholder engagement while designing, implementing and monitoring a study on diabetes and hypertension prevention in workplace settings in Nepal.

Methodology

We identified the stakeholders based on the 7P framework: Patients and public (clients), providers, payers, policy makers, product makers, principal investigators and purchasers. The identified stakeholders were engaged in prioritization of the research questions, planning data collection, designing, implementing and monitoring the intervention. Stakeholders were engaged through focus group discussions, in-depth interviews, participatory workshops, individual consultation, information sessions and representation in study team and implementation committees.

Results

The views of the stakeholders were synthesized in each step of the research process, from designing to interpreting the results. Stakeholder engagement helped to shape the methods and plan, and process for participant’s recruitment and data collection. In addition, it enhanced adherence to intervention, mutual learning, and smooth intervention adoption. The major

challenges were the time-consuming nature of the process, language barriers, and the differences in health and food beliefs between researchers and stakeholders.

Conclusion

It was possible to engage and benefit from stakeholder’s engagement on the design, implementation and monitoring of a workplace-based hypertension and diabetes management research program in Nepal.

INTRODUCTION

New scientific evidences on healthcare are generated in great volume [1]. However, much of this evidence has been difficult to implement in practice in real settings [2]. Stakeholder engagement can help to address this need by improving the relevance of research by increasing its transparency and accelerating the adoption into real world setting [3]. Stakeholder engagement is also important to generate knowledge, increase ownership, reduce conflict, encourage partnership, facilitate an inclusive decision making and promote equity [4].

Stakeholder engagement is promoted by health research funding organizations as well as researchers to achieve the desired goal [5]. Stakeholders can be engaged across stages of research including identifying topics, choosing hypotheses, analyzing data, and disseminating findings [6,7]. The levels of involvement range from consultation, to collaboration in bi-directional partnerships and to leading research projects [8].

Despite considerable attention to stakeholder engagement, there is limited information on how stakeholders are engaged. Some reviews describe the engagement in published literature and report impacts of engagement [9–12]. However, there is lack of reporting on how an engagement is implemented. It has been recommended that researchers systematically document and report information about engagement and its impact on individual projects [9,13]. Therefore, there is the need for demonstrating approaches that have been used to stakeholder engagement and opportunities to learn more about engagement in individual research projects. The purpose of this paper is to describe the process of stakeholder engagement and the lessons learned from this process while designing, implementing and monitoring a study on diabetes and hypertension prevention in workplace settings in Nepal.

MATERIALS AND METHODS

Study setting

Nepal Pioneer Worksite Intervention Study [14] started in 2016 at Dhulikhel Hospital-Kathmandu University Hospital in central Nepal with a goal to prevent diabetes and hypertension among the employees of Dhulikhel Hospital. Dhulikhel Hospital is an independently owned, not-for-profit institution which was conceived and supported by the Dhulikhel community. This study was registered on clincaltrials.gov (Identification Member:NCT03447340; Registration date: 27th February, 2018). The study was planned and executed in three phases:

Phase-1: Formative study

We conducted a formative study to gather data that is useful to develop and implement an intervention program to promote healthy eating at the worksite. The major aim of the formative study was appropriateness to culture and geography to develop meaningful interventions at individual and organizational level. The objective was to define and assess attributes of the study participants, understand the context, tailor intervention at local context, and build relationship between researcher, study populations and other stakeholders.

Phase-2: Develop culturally appropriate intervention and define research attributes

In the second phase, we used the information from the formative study to develop a culturally appropriate environmental level intervention to promote healthy eating in the cafeteria of the workplace. We further used this information to define study attributes such as primary outcomes, study design, implementation and monitoring strategies, and analysis plan.

Phase-3: Test the effectiveness of the intervention

In the third phase, we tested the effectiveness of the environmental level intervention using a two-step intervention study. In the first step, we assessed the effectiveness of a 6-month cafeteria intervention on cardio-metabolic risk using a pre-post design. In the second step, we conducted a 6-month, open-masked, two-arm randomized trial by allocating half of the participants to an individual behavioral intervention for the prevention of cardio-metabolic risk in order to estimate the additional effect of an individual level intervention. The design of the study has been published elsewhere[14].

Stakeholder engagement

Stakeholder definition

We defined stakeholder as an individual or group affected by or who can influence the environmental intervention to promote healthy eating in the workplace.

Engagement objectives

Our objectives of stakeholder engagement were (a) to identify the specific research needs in their worksite for promoting healthy eating, (b) to develop culturally appropriate healthy eating intervention; (c) to increase stakeholders' participation in study process such as determining study objectives and methods, describing study intervention, recruiting participants, implementing and monitoring the healthy eating intervention; (d) interpreting study results; and (e) enhancing sustainability.

Identification of stakeholders

We adopted the 7Ps framework [15] that identifies key groups to consider for engagement. The first, **patients and the public**, represented the current and potential consumers of patient-centered health care and population-focused public health. The second were **providers**, including individuals and organizations that provide care to patients and populations. **Purchasers**, the individuals and entities responsible for underwriting the costs of health care, such as employers, made up the third group. The fourth group consisted of **payers** who were responsible for reimbursement of medical care, such as insurers. The fifth composed of public **policy makers** and policy advocates working in the non-governmental sector. **Product makers**, representing drug and device manufacturers, comprised the sixth group, and **principal investigators,** or other researchers, made up the seventh. Since this was not a typical patient focus study, but rather a community-based prevention trial in an institutional setting, we modified the definition of the 7Ps according to our context. (Figure 1)

Figure 1: Stakeholder mapping for the Nepal Pioneer Worksite Intervention study to prevent cardiometabolic risk

Stakeholder engagement methods

We involved stakeholders in all three phase

At formative study phase

Formation of study team: After mapping out the stakeholder for the study, individuals representing each stakeholder category working in nutrition related research were purposively approached in-person and the study team was formed. We selected adult aged 18 years or older, employee of the hospital, who provided informed consent as stakeholder in each category. None of the stakeholders whom we approached declined to participate in the study. The stakeholders were represented as co-investigators and advisors.

Focus Group Discussions: We conducted four focus group discussions with 33 employees of the hospital to explore the current healthy eating environment, understand drivers and barriers for eating healthy, and to obtain their recommendations on improving environment to promote healthy eating at the hospital cafeteria.

The focus groups were conducted within the workplace setting in a private room to ensure confidentiality and open sharing of opinion. The FGDs were conducted in Nepali and audio taped after obtaining informed consent from the participants. The investigators, AS, or DT, moderated all FGDs in Nepali language and were assisted by a note-taker. In each session, a brief introduction of the study and ethical considerations about maintaining confidentiality of the participants, were explained. The moderator asked open-ended questions and probed for detail information. We used iterative process by discussing each FGD immediately after completion and suggesting necessary detail probing in emerging themes from finding.

In-depth interviews: We conducted 9 in-depth interviews with purposively selected hospital employees including a finance manager, a cafeteria manager, an administrative manager and 6 cafeteria operators after receiving informed consent. The investigators DT or AS conducted a semi-structured in-depth interviews in Nepali language using a pretested interview guide in a private room of the hospital. We used the iterative process for data collection. After each interview, we discussed each interview and identified the topics to be deeply explored into themes emerging in earlier interviews. All FGDs and interviews were transcribed verbatim into Nepali by trained research assistants. We used thematic analysis approach [16] to analyze the data.

At development of research design and intervention phase

Workshops: We conducted three iterative workshops with the representation of hospital administration, cafeteria managers, cafeteria staff, nutrition department, employee and researchers. The aims of the workshops were to consensually decide changes in cafeteria, clarify roles of each stakeholder and develop monitoring strategies. Each workshop was facilitated by DT, a co-investigator and an employee, using a workshop schedule and discussion guideline. The workshops took 3 hours. In the first hour, the facilitator explained the link between healthy eating and cardiometabolic risks. In the second hour, the findings from the formative study were shared in an interactive session, and in the third hour a guided discussion with the participants was conducted. The themes of [illegible] were: components of the healthy eating interventions, mode of interv[illegible] of implementation and monitoring, and method of evaluation - enables us to design effectiveness study. The stakeholders' in workshops provided inputs in decision making.

The last part of line 161 is not clear. Do you mean to enable us to plan an effective study?

We presented the initial findings with the stakeholders and collected their feedback in the interpretation of the results.

Food taste experiments: A major theme that emerged in focus group discussions, in-depth interviews and workshop included- acceptability of introducing whole grains in the cafeteria, especially brown rice. So, the research team decided and conducted a brown rice tasting experiment with 40 employees of the hospital. Brown rice tasting result showed that participant’s perspectives on brown rice changed after tasting. and the reason to switch was the higher quality of brown rice [17].

c) Individual consultation: The results of the formative study with further action plan were discussed individually with the hospital administrator, cafeteria manager, cafeteria chefs, nutritionist of the hospital and few employees of the hospital to get further feedback.

At testing of intervention phase

Meeting of study team: The study team met online every week for 30 to 60 minutes to discuss on a predetermined agenda and all decisions were taken jointly and recorded in written.

Information sessions: A 20-minute information session was conducted in all departments of the hospital to provide information regarding the study and collecting feedback. About 22 sessions were conducted with participation of 4 to 20 employees in each session. In the first five minutes, the study design was explained, the next 5 minutes were dedicated for the call for participation and expectations from the participants, and 10 minutes for discussion and feedback.

Formation of cafeteria intervention committee: A canteen intervention committee was formed comprising the representations of hospital administrator, human resource department, finance department, hygiene monitoring department, non-communicable disease prevention and management department, nutrition department, canteen managers, employee and researchers. This committee steered the development, implementation and monitoring of the cafeteria intervention. The committee made the plans for the cafeteria intervention, implementation and monitoring. It meets once a month to discuss the progress, problems and solutions.

Ethical consideration

We received approval from the institutional review board of Kathmandu University School of Medical Sciences, under Nepal Health Research Council (Reference number: 70/16) for the human subject contacts and obtained written informed consent from the participants before collecting data for research purpose.

RESULTS

Stakeholder identification

Based on the 7P framework, the identified stakeholders are presented in Figure 1. The identified stakeholders were customers or employees (patient and the public; purchasers); cafeteria managers, chef and staff (providers); hospital administration and finance department (payers; policy makers); Human resource department (policy maker); Cafeteria employee, bakery and Rice mills (Product maker); and Research team members (principal investigators).

Outcome of stakeholder engagement

Table 1 presents the stakeholder engagement in different activities within different study phases

Table 1: Stakeholder engagement in different activities within different study phases

**Study phase**	**Activity**	**Stakeholder involved**
**During Formative phase**	Formation of study team	• Principal investigator (Research team members)<br>• Purchasers (Employees including a medical doctor, laboratory personnel, a pharmacist and a community programs expert)<br>• Payers (The Hospital administrator)<br>• Policy maker (The Hospital administrator, A nutritionist)<br>• Provider (A cafeteria manager)
	Focus Group Discussions	• Principal investigator (Research team members)<br>• Purchasers (Employees)
	In-depth interviews	• Provider (Cafeteria operator, Cafeteria manager)<br>• Product maker (Cafeteria Staffs, Bakery and Rice mills)<br>• Policy maker (Human resource manager)
**During development of research**	Workshops	• Principal investigator (Research team members)<br>• Provider (Cafeteria operator, Cafeteria manager)<br>• Product maker (Cafeteria Staffs)<br>• Purchasers (Employees)

**design and intervention phase**	Individual consultation	• Principal investigator (Research team members)<br>• Purchasers (Employees including medical doctor, laboratory personnel, pharmacist and community programs expert)<br>• Payers (Hospital administrator)<br>• Policy maker (Hospital administrator, Nutritionist)<br>• Provider (Cafeteria operator, Cafeteria manager)
**During testing of intervention phase**	Meeting of study team	• Principal investigator (Research team members)<br>• Purchasers (Employees including a medical doctor, laboratory personnel, a pharmacist and a community programs expert)<br>• Payers (Hospital administrator)<br>• Policy maker (Hospital administrator, Nutritionist)<br>• Provider (Cafeteria operator, Cafeteria manager)
	Information sessions	• Principal investigator (Research team members)<br>• Policy makers (Departmental head and supervisors)<br>• Purchasers (Employees)
	Formation of cafeteria intervention committee:	• Product maker (Chef, Canteen operator)<br>• Policy makers (Administrative staffs, Nutritionist)<br>• Purchasers (employees including Medical doctors)

During formative study phase

Formation of study team: We formed a study team representing stakeholders from each category as investigator or an advisor. Investigators were those who directly participated in the day-to-day implementation of the study, compared to advisors who provided their suggestion and guidance when asked for [18]. Four employees - a medical doctor, laboratory personnel, a pharmacist and a community programs expert - joined the team as co-investigators. They brought their scientific expertise and unique perspective as a customer of cafeteria. The hospital administrator, a cafeteria manager and a nutritionist joined as advisor.

Focus Group Discussions: Majority of participants considered that the foods that were available in the hospital cafeteria were healthy. However, items such as confectionaries like cream donut, oily curry and meat, soda drinks, instant noodles etc. were considered unhealthy food. They emphasized that the foods were hygienically prepared and cheaper compared to hotels outside. Participants highlighted that the changes to improve food quality ~~will~~ be well received positively because most of them are health professionals. The most reported factors that would facilitate healthy eating were: (a) addition of healthy food options, (b) replacement of unhealthy foods with healthy options, and (c) appealing presentation of healthy foods. Active involvement of canteen management and administrator in the process of change was highly emphasized. The participants suggested to advertise healthy food options and educate both cafeteria operators and consumers on healthy eating. The major barriers to healthy eating were: (a) unavailability of healthy options, (b) lack of human resources to prepare healthy food, and (c) high price of healthy food and (d) food preferences. They suggested to add the healthy items which ~~will~~ demand less time and resources like adding the automated machines such as roti makers. The support staff commented that they might not be consuming the fruits even if they are added because of the high cost. However, health professionals expressed their willingness to pay more for access to healthy food. The participants were concerned that it would be difficult to change food preferences as the consumers prefer spicy and oily foods. Some of the unhealthy foods are greatly loved such as instant noodles, samosas, cream donuts, soda drinks etc., and changing food habit is challenging.

In-depth interviews: Cafeteria operator commented that the higher-level authorities should be involved in making healthy changes, deciding the menu and fixing the price. One of the

canteen managers highlighted the need of a committee to involve the canteen operator, administrative staff, medical doctors and nutritionist to decide on the change in food menu, price, and to monitor the availability of healthy options in canteen. The canteen operators mentioned that the hospital cafeteria provides food in subsidized price and does not intend to make profit. The operators were concerned about the lack of knowledge on healthy eating among the cafeteria staff and pointed that providing health education to them could facilitate making changes. They mentioned that the cafeteria staff receive training on hygiene occasionally but have never received training on healthy eating and healthy cooking. The major barrier for healthy eating reported by canteen operator were: (a) lack of adequate human resource to add food options; (b) lack of knowledge on the healthy cooking among cafeteria staff, (c) unavailability of healthy food options in the cafeteria and (d) no food supply for healthy foods such as brown rice, brown bread, organic vegetables etc. The canteen operator reported that they are not well-staffed to provide healthy foods such as whole wheat pan bread (roti), fruits and salad as their preparation is labor intensive. The human resource manager thought that increasing efficiency of the available staff was more important than adding staff. The stakeholders contributed to the interpretation of qualitative data and contextualize the findings. The details of the FGD and Interview results are available elsewhere [19]. In Brief, four focus group discussions of 33 participants and nine in-depth interviews among six canteen operators and three managers identified the availability of affordable healthy food options in the cafeterias, a commitment to such promotion by the cafeteria manager, operators, staff and hospital administration and the level of education of the employees, as the factors to promote healthy eating. In addition, the unavailability of healthy options, including the lack of food supply from local market, the

higher cost of healthy foods, individual food preferences and limited human resources in the cafeteria were identified as barriers for healthy eating [19].

During development of research design and intervention phase

Workshops: The discussions in the workshop were very interactive and productive. The cafeteria managers and cafeteria staffs emphasized the need for active involvement of the departments of finance and administration because these departments had major role deciding the menu and price of the food served in the cafeteria. This led to formation of canteen intervention committee with chairmanship of the hospital administrator and representatives from finance, human resource, nutrition, employee and researchers. There was univocal agreement on the need of adding healthy food items. However, lack of human resource in the cafeteria was a major challenge. In response, automated roti (whole wheat pan bread) maker was suggested. The details on which food to add and which to remove were discussed and finalized in the workshop. In addition, the pre-post design was suggested in contrast to randomized trial to test the effectiveness of the changes in cafeteria on cardiometabolic risk because it would be unethical to provide healthy food items in some cafeterias only. Additionally, the risk of contamination was emphasized as customers do not exclusively go to one cafeteria. Consumers and chefs raised the concern on acceptability of brown rice in place of white rice. Hence, a brown rice tasting experiment was suggested.

Individual consultation: Based on the findings of the FGDs, in-depth interviews and workshops, the study team prepared the description of the cafeteria intervention and proposed study design to test the effectiveness of the intervention on cardiometabolic risks. The proposal was described and discussed in-person with the stakeholders. Lack of

comparison group was indicated as a weakness of the study in the initial pre-post design. As a result, six months of control period was added before intervention was implemented to be able to compare outcome measures with and without the intervention.

During testing of intervention phase

Meeting of study team: In the weekly meeting of the study team, the implementation strategies were discussed and finalized in details. This included details on disseminating information on study, call for participation, informed consent administration, data collection from the participants, enrolment planning, and intervention start dates. The internal insights of employees in the study team members guided the decision making. When the effectiveness study started, the progress on enrollment, problems and strategies were discussed weekly. For example, when there was slow blood sample collection, the representative from the laboratory suggested to provide breakfast voucher to the participants. Adding breakfast voucher worth 70 cents in subsequent blood draw appointment speeded up the sample collection. Similarly, it was difficult to enroll the nurses who had night duties. The doctor in the study team suggested to consider nurse's duty roster and contact nurses immediately after completion of duty hours in the morning. This strategy helped to increase the participation of the nurses in the study.

Information sessions: The information session helped to reach and interact with wider employee group. The department head provided date and venue for the information session. During the information session, feedback was obtained on the design of the study and participant recruitment. A major issue that came up was the involvement of supervisors in participants' recruitment. The employees commented that presence of supervisors during the

information session might be perceived as encouragement from them to participate. To make the participation voluntary, we conducted the sessions in absence of higher-level staff in each department, the identifiers of the participants were collected separately and were not disclosed to anyone.

Formation of cafeteria intervention committee: The cafeteria intervention committee had key decisive role in designing, implementing, monitoring and evaluating the cafeteria intervention. Each component of the intervention was discussed and decided by the intervention committee after weighing their benefits, financial implications and perceived acceptance by the employee. The committee developed a monitoring sheet that the nutritionist and a research team member filled every week to measure the adherence to the defined intervention in all four cafeterias. The monitoring team also interacted with the chefs, cafeteria staffs and customers informally and collected their concerns. The monitoring data were assessed and concerns were presented to the committee every month. The committee discussed the problems and strategies to address them. For example, in the initial week, there was problem of supply of whole grains such as brown rice and brown bread. The committee approached the local mill and a local bakery and ordered brown rice and brown bread for the cafeteria. After adding fruits on the menu, the sales of fruits in the first two months were low. The committee decided to launch a week long campaign to promote fruits in each cafeteria which boosted their sales.

Benefits and challenges of stakeholder engagement

We are confident that our project, the project goals, methods and intervention have evolved positively based on our collaborations with stakeholders. Most importantly, their input was

necessary in determining and describing cafeteria intervention. Their continuous involvement in monitoring helped in solving problems promptly and enhanced adherence to intervention. In addition, their input shaped methods, plans and processes for recruiting participants and data collection.

While we had a positive experience of involving stakeholder, it was not devoid of challenges. A major challenge was time availability of stakeholders as they were busy in their respective works. To address this, we scheduled meetings according to the availability of the stakeholders. We had to conduct multiple workshops and information sessions to accommodate the time of cafeteria staff and hospital staff. This was time and resource consuming. It was also important to allow time for stakeholders to get acquainted with their new roles associated with intervention. Their knowledge of a healthy diet was dominated by the cultural norms and there was some misconception. So, we invested time and resources in training them on healthy eating. This also led to initiation of positive changes in cafeterias during the control period before the predetermined intervention date. We also felt that research priorities do not necessarily meet the priorities of the stakeholders. For example, the cafeteria staff emphasized more on the changes in physical structures such as adding culinary. There was language barrier, as researchers were comfortable using scientific jargons such as ‘epidemiology’ ‘control’ ‘confounding’, ‘subjects’ etc. making the stakeholders confused at times. It was difficult to translate or interpret these works well in local language. Additionally, the food beliefs of researchers and stakeholders were different. Stakeholders strongly believe in eating warm ‘white rice’ twice a day. In addition, fruits were not considered appropriate to take during lunch. Knowledge sessions on healthy diets addressed these deep rooted beliefs to some extent but changing food belief system takes longer time and more effort [6,20].

DISCUSSION

We identified employees, cafeteria managers, workplace administrators, finance manager, human resource manager, cafeteria staff and researchers as major stakeholders in the Nepal pioneer worksite intervention study to prevent cardiometabolic risks. The stakeholders were included in the study team and they brought their expertise in the subject matter as well as insights as employees and customers of the cafeteria. The exploration of the facilitators and barriers to healthy eating at their worksite provided a base for defining the intervention and study attributes. Interactive workshops provided opportunity and mandate for the stakeholders to participate in decision making and strengthened the investigator-stakeholder relationship. Individual consultations helped get stronger buy-in and commitment from the higher authorities and employees. Regular meeting of the study team and canteen intervention committee guaranteed continuous engagement in problem solving and decision making. It reinforced ownership and enhanced project sustainability. The major challenges were the time-consuming nature of the process, language barriers, and differences in health and food beliefs between researchers and stakeholders.

We mapped and engaged 7 different types of stakeholders in our study. Patient-centered outcome research projects have reported engaging clinicians, caregivers, advocacy organizations and health system representatives; but only few engaged payers and policy makers [13,21]. We incorporated the views of multiple types of stakeholders into our study during formative phase, development of intervention and study and execution of the study. For example, we formed cafeteria intervention committee based on the suggestion of cafeteria manager in in-depth interview. Brown-rice tasting experiment was also conducted to solve the concern of consumers and chefs on the acceptability of brown rice ~~in workshop.~~ Engagement has most frequently been reported in developing research

questions [9]. This study described the effort, attention, resources and flexibility for concerted engagement emphasizing on meaningful and continuous collaboration, shared leadership and decision-making power, and adaptation to the needs of stakeholders. Other researchers have highlighted the importance of sharing power for successful research engagement [22].

The input from different stakeholder influenced our study goals, intervention, methods and materials. This enhanced the appropriateness and relevance in the local context. Similar experience was reported by other investigators [6]. Making research more beneficiaries-centric, culturally relevant and accessible to potential research participants have been reported to refine proposals, recruitment materials, and participant compensation [23]. In our project, the stakeholders contributed to the interpretation of qualitative data and contextualize the findings, similar to the study by Boote J et al [11]. This is consistent with the available evidence on positive impacts of engaged research [6,12].

The stakeholder engagement has contributed positively in our study from formative phase to execution. Various levels of stakeholder involvement, such as consultation, collaboration or user-control has been proposed [24]. However, caution has been recommended against assuming that involving higher levels is better [25]. Some researchers in a national survey from UK claimed that decisions about the appropriateness of consumer involvement in research should be made on a case-by-case basis [13].

The major challenges that we faced were the time-consuming nature of the process, language barriers, and differences in health and food beliefs between researchers and stakeholders. Similar challenges have been reported in other studies [6,20]. Lack of time—both for stakeholders and researchers—was the most frequently noted challenge and was identified as a top barrier to

engagement in a recent survey of investigators [20]. Thus, it is crucial to develop strategies that maximize stakeholder input with minimum time and resources [26].

The generalizability of our findings is limited because of the unique setting where the research was conducted. Because it was a university hospital, we had an advantage of including the employees with specific research expertise as co-investigators. The stakeholders had more interest in and experience with the health interventions and research. Thus, their knowledge and attitude about engagement may be more positive.

CONCLUSION

It was possible to engage and benefit from stakeholder's engagement on the design, implementation and monitoring of a workplace-based hypertension and diabetes management research program in Nepal. We recommend ongoing program development and implementation by stakeholder input. In future, we plan to evaluate the influence of stakeholders on the research project and assess the engagement and effectiveness through their perspective, as well as longer term outcomes on health decision making beyond the conduction of research.

ACKNOWLEDGMENTS

We would like to acknowledge the Dhulikhel Hospital – Kathmandu University Hospital for their permission and cooperation to conduct this study. We appreciate the contribution of all the study participants.

AUTHOR CONTRIBUTIONS

Conceptualization: Archana Shrestha (AS), Dipesh Tamrakar(DT), Donna Spiegelman (DS)

Formal analysis: Bhawana Shrestha (BS), Biraj Man Karmacharya(BMK), Abha Shrestha (AbS), Prajjwal Pyakurel(PP)

Writing – original draft: Archana Shrestha (AS)

Writing – review & editing: Archana Shrestha (AS), Dipesh Tamrakar (DT), Bhawana Shrestha (BS), Biraj Man Karmacharya(BMK), Abha Shrestha(AbS), Prajjwal Pyakurel(PP), Donna Spiegelman (DS)

REFERENCES

1. Titler MG. The Evidence for Evidence-Based Practice Implementation. In: Hughes RG, editor. Patient Safety and Quality: An Evidence-Based Handbook for Nurses. Rockville (MD): Agency for Healthcare Research and Quality (US); 2008. Available: http://www.ncbi.nlm.nih.gov/books/NBK2659/

2. Zilberberg MD. The clinical research enterprise: time to change course? Jama. 2011;305: 604–605.

3. Ray KN, Miller E. Strengthening stakeholder-engaged research and research on stakeholder engagement. Journal of comparative effectiveness research. 2017;6: 375–389.

4. Mathur VN, Price ADF, Austin S. Conceptualizing stakeholder engagement in the context of sustainability and its assessment. Construction Management and Economics. 2008;26: 601–609. doi:10.1080/01446190802061233

5. Kok MO, Gyapong JO, Wolffers I, Ofori-Adjei D, Ruitenberg J. Which health research gets used and why? An empirical analysis of 30 cases. Health research policy and systems. 2016;14: 1–18.

6. Shippee ND, Domecq Garces JP, Prutsky Lopez GJ, Wang Z, Elraiyah TA, Nabhan M, et al. Patient and service user engagement in research: a systematic review and synthesized framework. Health Expectations. 2015;18: 1151–1166.

7. Mullins CD, Abdulhalim AM, Lavallee DC. Continuous patient engagement in comparative effectiveness research. Jama. 2012;307: 1587–1588.

8. Lowes L, Hulatt I. Involving service users in health and social care research. Routledge; 2013.

9. Concannon TW, Fuster M, Saunders T, Patel K, Wong JB, Leslie LK, et al. A systematic review of stakeholder engagement in comparative effectiveness and patient-centered outcomes research. Journal of general internal medicine. 2014;29: 1692–1701.

10. Forsythe LP, Szydlowski V, Murad MH, Ip S, Wang Z, Elraiyah TA, et al. A systematic review of approaches for engaging patients for research on rare diseases. Journal of general internal medicine. 2014;29: 788–800.

11. Boote J, Baird W, Sutton A. Involving the public in systematic reviews: a narrative review of organizational approaches and eight case examples. Journal of comparative effectiveness research. 2012;1: 409–420.

12. Brett J, Staniszewska S, Mockford C, Herron- Marx S, Hughes J, Tysall C, et al. Mapping the impact of patient and public involvement on health and social care research: a systematic review. Health expectations. 2014;17: 637–650.

13. Barber R, Boote JD, Cooper CL. Involving consumers successfully in NHS research: a national survey. Health Expectations. 2007;10: 380–391.

14. Shrestha A, Tamrakar D, Karmacharya BM, Shrestha A, Shrestha R, Bhatta RD, et al. Nepal Pioneer Worksite Intervention Study to lower cardio-metabolic risk factors: design and protocol. BMC cardiovascular disorders. 2019;19: 1–10.

15. Concannon TW, Meissner P, Grunbaum JA, McElwee N, Guise J-M, Santa J, et al. A new taxonomy for stakeholder engagement in patient-centered outcomes research. Journal of general internal medicine. 2012;27: 985–991.

16. Boyatzis RE. Transforming qualitative information: Thematic analysis and code development. sage; 1998.

17. Gyawali P, Tamrakar D, Shrestha A, Shrestha H, Karmacharya S, Bhattarai S, et al. Consumer acceptance and preference for brown rice—A mixed- method qualitative study from Nepal. Food Science & Nutrition. 2022; fsn3.2803. doi:10.1002/fsn3.2803

18. Sciences NA of, Science NA of E (US) and I of M (US) C on, Engineering, Policy and P. ADVISING AND MENTORING. On Being a Scientist: A Guide to Responsible Conduct in Research: Third Edition. National Academies Press (US); 2009. Available: https://www.ncbi.nlm.nih.gov/books/NBK214555/

19. Tamrakar D, Shrestha A, Rai A, Karmacharya BM, Malik V, Mattei J, et al. Drivers of healthy eating in a workplace in Nepal: a qualitative study. BMJ Open. 2020;10: e031404. doi:10.1136/bmjopen-2019-031404

20. Boote J, Baird W, Beecroft C. Public involvement at the design stage of primary health research: a narrative review of case examples. Health policy. 2010;95: 10–23.

21. Forsythe LP, Ellis LE, Edmundson L, Sabharwal R, Rein A, Konopka K, et al. Patient and stakeholder engagement in the PCORI pilot projects: description and lessons learned. Journal of general internal medicine. 2016;31: 13–21.

22. Westfall JM, Fagnan LJ, Handley M, Salsberg J, McGinnis P, Zittleman LK, et al. Practice-based research is community engagement. The Journal of the American Board of Family Medicine. 2009;22: 423–427.

23. Joosten YA, Israel TL, Williams NA, Boone LR, Schlundt DG, Mouton CP, et al. Community engagement studios: a structured approach to obtaining meaningful input from stakeholders to inform research. Academic Medicine. 2015;90: 1646.

24. Dent H. Involving the Public in NHS, Public Health and Social Care Research: Briefing notes for researchers. Nurse Researcher. 2005;12: 89–91.

25. Barnes M, Cotterell P. Critical and different perspectives on user involvement. Critical perspectives on user involvement. 2012; 225–33.

26. Frank L, Forsythe L, Ellis L, Schrandt S, Sheridan S, Gerson J, et al. Conceptual and practical foundations of patient engagement in research at the patient-centered outcomes research institute. Quality of Life Research. 2015;24: 1033–1041.

Figure 1: Stakeholder mapping for the Nepal Pioneer Worksite Intervention study to prevent cardiometabolic risk

Click here to access/download;Figure;Figure 1.png

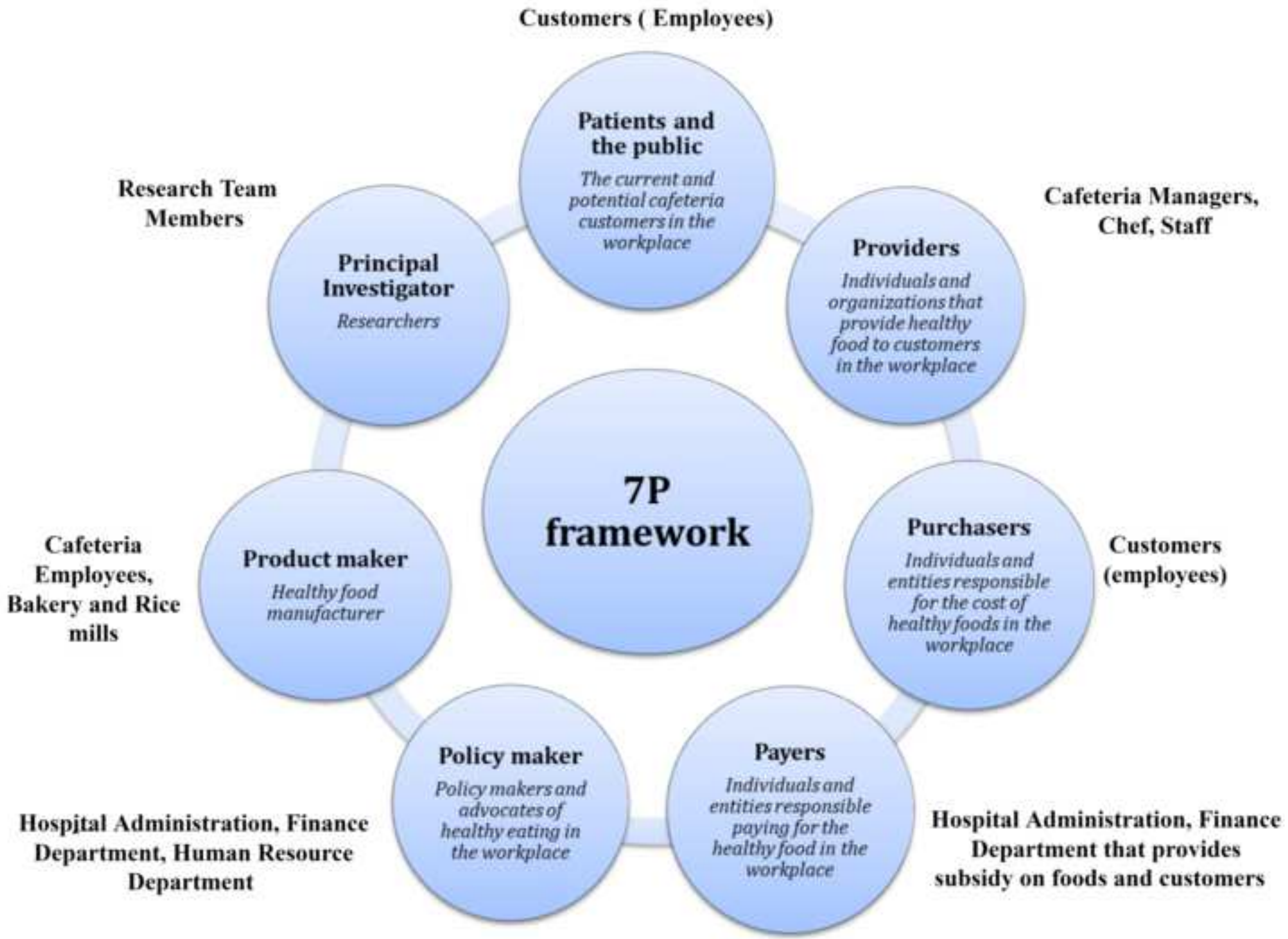

Stakeholder engagement in a hypertension and diabetes prevention research program – ~~Description and Lessons learned~~

Archana Shrestha[1,2*], Dipesh Tamrakar[1], Bhawana Shrestha[1], Biraj Man Karmacharya[1], Abha Shrestha[1], Prajjwal Pyakurel[2], Donna Spiegelman[3]

[1] Department of Public Health, Kathmandu University School of Medical Sciences, Dhulikhel, Nepal

[2] Department of Community Medicine, BP Koirala Institute of Health Sciences, Dharan, Nepal

[3] Department of Biostatistics, Yale School of Public Health, New Haven, USA

* Corresponding author: Dr. Archana Shrestha; E-mail: deararchana@gmail.com

Abstract

Background

Stakeholder engagement is important from the management point of view to capture knowledge, increase ownership, reduce conflict, encourage partnership, as well as to develop an ethical perspective that facilitates inclusive decision making and promotes equity. However, there is dearth of literature in the process of stakeholder engagement. The purpose of this paper is to describe the process of increasing stakeholder engagement and highlight the lessons learnt on stakeholder engagement while designing, implementing and monitoring a study on diabetes and hypertension prevention in workplace settings in Nepal.

Methodology

We identified the stakeholders based on the 7P framework: Patients and public (clients), providers, payers, policy makers, product makers, principal investigators and purchasers. The identified stakeholders were engaged in prioritization of the research questions, planning data collection, designing, implementing and monitoring the intervention. Stakeholders were engaged through focus group discussions, in-depth interviews, participatory workshops, individual consultation, information sessions and representation in study team and implementation committees.

Results

The views of the stakeholders were synthesized in each step of the research process, from designing to interpreting the results. Stakeholder engagement helped to shape the methods and plan, and process for participant’s recruitment and data collection. In addition, –it enhanced adherence to intervention, mutual learning, and smooth intervention adoption. The major

challenges were the time-consuming nature of the process, language barriers, and the differences in health and food beliefs between researchers and stakeholders.

Conclusion

It was possible to engage and benefit from stakeholder's engagement on the design, implementation and monitoring of a workplace-based hypertension and diabetes management research program in Nepal.

INTRODUCTION

New scientific evidences on healthcare is are produced generated in great volume[1] [1]. However, much of this evidence has been difficult to implement in practice in real settings[2]. Stakeholder engagement can help to address this need by improving the relevance of research by increasing its transparency and accelerating the adoption into real world setting[3]. Stakeholder engagement is also important to generate knowledge, increase ownership, reduce conflict, encourage partnership, facilitate an inclusive decision making and promote equity[4].

Stakeholder engagement is promoted by health research funding organizations as well as researchers to achieve the desired goal[5]. Stakeholders can be engaged across stages of research including identifying topics, choosing hypotheses, analyzing data, and disseminating findings[6,7]. The levels of involvement range from consultation, to collaboration in bi-directional partnerships and to leading research projects[8].

Despite considerable attention to stakeholder engagement, there is limited information on how stakeholders are engaged. Some reviews describe the engagement in published literature and report impacts of engagement [9–12]. However, there is lack of reporting on how an engagement is implemented. It has been recommended that researchers systematically document and report information about engagement and its impact on individual projects[9,13]. Therefore, there is the need for demonstrating approaches that have been used to stakeholder engagement and opportunities to learn more about engagement in individual research projects. The purpose of this paper is to describe the process of stakeholder engagement and the lessons learned from this process while designing, implementing and monitoring a study on diabetes and hypertension prevention in workplace settings in Nepal.

MATERIALS AND METHODS

Study setting

Nepal Pioneer Worksite Intervention Study [14] started in 2016 at Dhulikhel Hospital-Kathmandu University Hospital in central Nepal with a goal to prevent diabetes and hypertension among the employees of Dhulikhel Hospital. Dhulikhel Hospital is an independently owned, not-for-profit institution which was conceived and supported by the Dhulikhel community. This study was registered on clincaltrials.gov (Identification Member:NCT03447340; Registration date: 27th February, 2018). The study was planned and executed in three phases:

Phase-1: Formative study

We conducted a formative study to gather data that is useful to develop and implement an intervention program to promote healthy eating at the worksite. The major aim of the formative study was appropriateness to culture and geography to develop meaningful interventions at individual and organizational level. The objective was to define and assess attributes of the study participants, understand the context, tailor intervention at local context, and build relationship between researcher, study populations and other stakeholders.

Phase-2: Develop culturally appropriate intervention and define research attributes

In the second phase, we used the information from the formative study to develop a culturally appropriate environmental level intervention to promote healthy eating in the cafeteria of the workplace. We further used this information to define study attributes such as primary outcomes, study design, implementation and monitoring strategies, and analysis plan.

Phase-3: Test the effectiveness of the intervention

In the third phase, we tested the effectiveness of the environmental level intervention using a two-step intervention study. In the first step, we assessed the effectiveness of a 6-month cafeteria intervention on cardio-metabolic risk using a pre-post design. In the second step, we conducted a 6-month, open-masked, two-arm randomized trial by allocating half of the participants to an individual behavioral intervention for the prevention of cardio-metabolic risk in order to estimate the additional effect of an individual level intervention. The design of the study has been published elsewhere[14].

Stakeholder engagement

Stakeholder definition

We defined stakeholder as an individual or group affected by or who can influence the environmental intervention to promote healthy eating in the workplace.

Engagement objectives

Our objectives of stakeholder engagement were (a) to identify the specific research needs in their worksite for promoting healthy eating, (b) to develop culturally appropriate healthy eating intervention; (c) to increase stakeholders' participation in study process such as determining study objectives and methods, describing study intervention, recruiting participants, implementing and monitoring the healthy eating intervention; (d) interpreting study results; and (e) enhancing sustainability.

Identification of stakeholders

We adopted the 7Ps framework[15] that identifies key groups to consider for engagement. The first, **patients and the public**, represented the current and potential consumers of patient-centered health care and population-focused public health. The second were **providers**, including individuals and organizations that provide care to patients and populations. **Purchasers**, the individuals and entities responsible for underwriting the costs of health care, such as employers, made up the third group. The fourth group consisted of **payers** who were responsible for reimbursement of medical care, such as insurers. The fifth composed of public **policy makers** and policy advocates working in the non-governmental sector. **Product makers**, representing drug and device manufacturers, comprised the sixth group, and **principal investigators,** or other researchers, made up the seventh. Since this was not a typical patient focus study, but rather a community-based prevention trial in an institutional setting, we modified the definition of the 7Ps according to our context. (Figure 1)

Figure 1: Stakeholder mapping for the Nepal Pioneer Worksite Intervention study to prevent cardiometabolic risk

Stakeholder engagement methods

We involved stakeholders in all three phase~~s.~~

At formative study phase

Formation of study team: After mapping out the stakeholder for the study, individuals representing each stakeholder category working in nutrition related research were purposively approached ~~individually~~ in-person and the study team was formed. We selected adult aged 18 years or older, employee of the hospital, who provided informed consent as stakeholder in each category~~.~~ None of the stakeholders whom we approached declined to participate in the study. The stakeholders were represented as co-investigators and advisors.

Focus Group Discussions: We conducted four focus group discussions with 33 employees of the hospital to explore the current healthy eating environment, understand drivers and barriers for eating healthy, and to obtain their recommendations on improving environment to promote healthy eating at the hospital cafeteria.

The focus groups were conducted within the workplace setting in a private room to ensure confidentiality and open sharing of opinion. The FGDs were conducted in Nepali and audio taped after obtaining informed consent from the participants. The investigators, AS, or DT, moderated all FGDs in Nepali language and were assisted by a note-taker. In each session, a brief introduction of the study and ethical considerations about maintaining confidentiality of the participants, were explained. The moderator asked open-ended questions and probed for detail information. We used iterative process by discussing each FGD immediately after completion and suggesting necessary detail probing in emerging themes from finding.

In-depth interviews: We conducted 9 in-depth interviews with purposively selected hospital employees including a finance manager, a cafeteria manager, an administrative manager and 6 cafeteria operators after receiving informed consent. The investigators DT or AS conducted a semi-structured in-depth iInterviews in Nepali language using a pretested interview guide in a private room of the hospital. We used the iterative process for data collection. After each interview, we discussed each interview and identified the topics to be deeply explored into themes emerging in earlier interviews. All FGDs and interviews were transcribed verbatim into Nepali by trained research assistants. We used thematic analysis approach [16] to analyze the data.

At development of research design and intervention phase

Workshops: We conducted three iterative workshops with the representation of hospital administration, cafeteria managers, cafeteria staff, nutrition department, employee and researchers. . The aims of the workshops were to consensually decide changes in cafeteria, clarify roles of each stakeholder and develop monitoring strategies. Each workshop was facilitated by DT, a co-investigator and an employee, using a workshop schedule and discussion guideline. The workshops took 3 hours. In the first hour, the facilitator explained the link between healthy eating and cardiometabolic risks. In the second hour, the findings from the formative study were shared in an interactive session, and in the third hour a guided discussion with the participants was conducted. The themes of the guided discussion were: components of the healthy eating interventions, mode of intervention delivery, mode of implementation and monitoring, and method of evaluation - that led toenables us to design of the effectiveness study. The stakeholders' in workshops provided inputs in

Formatted: Not Highlight

decision making. We presented the initial findings with the stakeholders and collected their feedback in the interpretation of the results.

Food taste experiments: A major theme that emerged in focus group discussions, in-depth interviews and workshop ~~was regarding~~ included- acceptability of introducing whole grains in the cafeteria, especially brown rice. ~~So, we~~ So, the research team decided and ~~-~~conducted a brown rice tasting experiment with 40 employees of the hospital. Brown rice tasting result showed that ~~P~~participant's perspectives on brown rice ~~was found to be~~ changed after tasting. ~~Participants~~ and the reason to~~were willing to~~ switch ~~to brown rice as they found that brown rice had~~ was the higher quality of brown rice.[17]

c) Individual consultation: The results of the formative study with further action plan were discussed individually with the hospital administrator, cafeteria manager, cafeteria chefs, nutritionist of the hospital and few employees of the hospital to get further feedback.

At testing of intervention phase

Meeting of study team: The study team met online every week for 30 to 60 minutes to discuss on a predetermined agenda and all decisions were taken jointly and recorded in written.

Information sessions: A 20-minute information session was conducted in all departments of the hospital to provide information regarding the study and collecting feedback. About 22 sessions were conducted with participation of 4 to 20 employees in each session. In the first five minutes, the study design was explained, the next 5 minutes were dedicated for the call for participation and expectations from the participants, and 10 minutes for discussion and feedback.

Formation of cafeteria intervention committee: A canteen intervention committee was formed~~–~~ ~~the~~comprising the representations of hospital administrator, human resource department, finance department, hygiene monitoring department, non-communicable disease prevention and management department, nutrition department, canteen managers, employee and researchers. This committee steered the development, implementation and monitoring of the cafeteria intervention. The committee made the plans for the cafeteria intervention, implementation and monitoring. It meets once a month to discuss ~~about~~ the progress, problems and solutions.

Ethical consideration

We received approval from the institutional review board of Kathmandu University School of Medical Sciences, under ~~and~~ Nepal Health Research Council (Reference number: 70/16) for the human subject contacts and obtained written informed consent from the participants before collecting data for research purpose.

RESULTS

Stakeholder identification

Based on the 7P framework, the identified stakeholders are presented in Figure 1. The identified stakeholders were- customers or employees (patient and the public; purchasers); cafeteria managers, chef and staff (providers); hospital administration and finance department (payers; policy makers); Human resource department (policy maker); Cafeteria employee, bakery and Rice mills (Product maker); and Research team members (principal investigators).

Outcome of stakeholder engagement

Table 1 presents the stakeholder engagement in different activities within different study phases

Table 1: Stakeholder engagement in different activities within different study phases

Study phase	Activity	Stakeholder involved
**During Formative phase**	Formation of study team	• Principal investigator (Research team members)<br>• Purchasers (Employees -including a medical doctor, a laboratory personnel, a pharmacist and a community programs expert)<br>• Payers (The Hospital administrator)<br>• Policy maker (The Hospital administrator, A nutritionist)<br>• Provider (A cafeteria manager)
	Focus Group Discussions	• Principal investigator (Research team members)<br>• Purchasers (Employees))
	In-depth interviews	• Provider (Cafeteria operator, Cafeteria manager)<br>• Product maker (Cafeteria Staffs, Bakery and Rice mills)<br>• Policy maker (Human resource manager)
**During development of research**	Workshops	• Principal investigator (Research team members)<br>• Provider (Cafeteria operator, Cafeteria manager)<br>• Product maker (Cafeteria Staffs),<br>• Purchasers (Employees)

**design and intervention phase**	Individual consultation	• Principal investigator (Research team members)<br>• Purchasers (~~-~~Employees including medical doctor, laboratory personnel, pharmacist and community programs expert)<br>• Payers (Hospital administrator)<br>• Policy maker (Hospital administrator, Nutritionist)<br>• Provider (Cafeteria operator, Cafeteria manager)
**During testing of intervention phase**	Meeting of study team	• Principal investigator (Research team members)<br>• Purchasers (Employees including a medical doctor, ~~a laboratory personnel~~laboratory personnel, a pharmacist and a community programs expert)<br>• Payers (Hospital administrator)<br>• Policy maker (Hospital administrator, Nutritionist)<br>• Provider (Cafeteria operator, Cafeteria manager)
	Information sessions	• Principal investigator (Research team members)<br>• Policy makers (Departmental head and supervisors)<br>• Purchasers (Employees)
	Formation of cafeteria intervention committee:	• Product maker (Chef, Canteen operator)<br>• Policy makers (Administrative staffs, Nutritionist)<br>• Purchasers (employees including Medical doctors)

During formative study phase

Formation of study team: We formed a study team representing stakeholders from each category as investigator or an advisor. Investigators were those who directly participated in the day-to-day implementation of the study, compared to advisors who provided their suggestion and guidance when asked for [18]. Four employees - a medical doctor, ~~a~~ laboratory personnel, a pharmacist and a community programs expert - joined the team as co-investigators. They brought their scientific expertise and unique perspective as a customer of cafeteria. The hospital administrator, a cafeteria manager and a nutritionist joined as advisor.

Focus Group Discussions: Majority of participants considered that the foods that were available in the hospital cafeteria were healthy. However, items such as confectionaries like cream donut, oily curry and meat, soda drinks, instant noodles etc. were considered unhealthy food. They emphasized that the foods were hygienically prepared and cheaper compared to hotels outside. Participants highlighted that the changes to improve food quality will be well received positively because most of them are health professionals. The most reported factors that would facilitate healthy eating were: (a) addition of healthy food options, (b) replacement of unhealthy foods with healthy options, and (c) appealing presentation of healthy foods. Active involvement of canteen management and administrator in the process of change was highly emphasized. The participants suggested to advertise healthy food options and educate both cafeteria operators and consumers on healthy eating. The major barriers to healthy eating were: (a) unavailability of healthy options, (b) lack of human resources to prepare healthy food, and (c) high price of healthy food and (d) food preferences. They suggested to add the healthy items which will demand less time and resources like adding the automated machines such as roti makers. The support staff commented that they might not be consuming the fruits even if they are added because of the high cost. However, health professionals expressed their willingness to pay more for access to healthy food. The participants were concerned that it would be difficult to change food preferences as the consumers prefer spicy and oily foods. Some of the unhealthy foods are greatly loved such as instant noodles, samosas, cream donuts, soda drinks etc., and changing food habit is challenging.

In-depth interviews: Cafeteria operator commented that the higher-level authorities should be involved in making healthy changes, deciding the menu and fixing the price. One of the

Formatted: Indent: Left: 0.38", Line spacing: Double

canteen managers highlighted the need of a committee to involve~~ing~~ the canteen operator, administrative staff, medical doctors and nutritionist to decide on the change in food menu, price, and to monitor the availability of healthy options in canteen. The canteen operators mentioned that the hospital cafeteria provides food in subsidized price and does not intend to make profit. The operators were concerned about the lack of knowledge on healthy eating among the cafeteria staff and pointed that providing health education to them could facilitate making changes. They mentioned that the cafeteria staff receive training on hygiene occasionally but have never received training on healthy eating and healthy cooking. The major barrier for healthy eating reported by canteen operator were: (a) lack of adequate human resource to add food options; (b) lack of knowledge on the healthy cooking among cafeteria staff, (c) unavailability of healthy food options in the cafeteria and (d) no food supply for healthy foods such as brown rice, brown bread, organic vegetables etc. The canteen operator reported that they are not well-staffed to provide healthy foods such as whole wheat pan bread (roti), fruits and salad as their preparation is labor intensive. The human resource manager thought that increasing efficiency of the available staff was more important than adding staff. The stakeholders contributed to the interpretation of qualitative data and contextualize the findings. The details of the FGD and Interview results are available elsewhere [19]. In Brief, four focus group discussions of 33 participants and nine in-depth interviews among six canteen operators and three managers identified the availability of affordable healthy food options in the cafeterias, a commitment to such promotion by the cafeteria manager, operators, staff and hospital administration and the level of education of the employees, as the factors to promote healthy eating. In addition, the unavailability of healthy options, including the lack of food supply from local market, the higher cost of healthy foods,

Formatted: Not Highlight

Formatted: Not Highlight

individual food preferences and limited human resources in the cafeteria were identified as barriers for healthy eating.[19]

During development of research design and intervention phase

Workshops: The discussions in the workshop were very interactive and productive. The cafeteria managers and cafeteria staffs emphasized the need for active involvement of the departments of finance and administration because these departments had major role deciding the menu and price of the food served in the cafeteria. This led to formation of canteen intervention committee with chairmanship of the hospital administrator and representatives from finance, human resource, nutrition, employee and researchers. There was univocal agreement on ~~in~~ the need of adding healthy food items. However, lack of human resource in the cafeteria was a major challenge. In response, automated roti (whole wheat pan bread) maker was suggested. The details on which food to add and which to remove were discussed and finalized in the workshop. In addition, the pre-post design was suggested in contrast to randomized trial to test the effectiveness of the changes in cafeteria on cardiometabolic risk because it would be unethical to provide healthy food items in some cafeterias only. Additionally, the risk of contamination was emphasized as customers do not exclusively go to one cafeteria. Consumers and chefs raised the concern on acceptability of brown rice in place of white rice. Hence, a brown rice tasting experiment was suggested.

Individual consultation: Based on the findings of the FGDs, in-depth interviews and workshops, the study team prepared the description of the cafeteria intervention and proposed study design to test the effectiveness of the intervention on cardiometabolic risks.

The proposal was described and discussed in-person with the stakeholders. Lack of comparison group was indicated as a weakness of the study in the initial pre-post design. As a result, six months of control period was added before intervention was implemented to be able to compare outcome measures with and without the intervention.

Formatted: Indent: Left: 0"

During testing of intervention phase

Meeting of study team: In the weekly meeting of the study team, the implementation strategies were discussed and finalized in ~~minute~~ details. This included details on disseminating information on study, call for participation, informed consent administration, data collection from the participants, enrolment planning, and intervention start dates. The internal insights of employees in the study team members guided the decision making. When the effectiveness study started, the progress on enrollment, problems and strategies were discussed weekly. For example, when there was slow blood sample collection, the representative from the laboratory suggested to provide breakfast voucher to the participants. Adding breakfast voucher worth 70 cents in subsequent blood draw appointment speeded up the sample collection. Similarly, it was difficult to enroll the nurses who had night duties. The doctor in the study team suggested to consider ~~the~~ nurse's duty roster and ~~suggested to~~ contact ~~them~~ nurses immediately after ~~their~~ completion of duty ~~is over~~hours in the morning. This strategy helped to increase the participation of the nurses in the study.

Information sessions: The information session helped to reach and interact with wider employee group. The department head provided date and venue for the information session. During the information session, feedback was obtained on the design of the study and participant recruitment. A major issue that came up was the involvement of supervisors in

participants’ recruitment. The employees commented that presence of supervisors during the information session might be perceived as encouragement from them to participate. To make the participation voluntary, we conducted the sessions in absence of higher-level staff in each department, the identifiers of the participants were collected separately and were not disclosed to anyone.

Formation of cafeteria intervention committee: The cafeteria intervention committee had key decisive role in designing, implementing, monitoring and evaluating the cafeteria intervention. Each component of the intervention was discussed and decided by the intervention committee after weighing their benefits, financial implications and perceived acceptance by the employee. The committee developed a monitoring sheet that the nutritionist and a research team member filled every week to measure the adherence to the defined intervention in all four cafeterias. The monitoring team also interacted with the chefs, cafeteria staffs and customers informally and collected their concerns. The monitoring data were assessed and concerns were presented to the committee every month. The committee discussed the problems and strategies to address them. For example, in the initial week, there was problem of supply of whole grains such as brown rice and brown bread. The committee approached the local mill and a local bakery and ordered brown rice and brown bread for the cafeteria. After adding fruits on the menu, the sales of fruits in the first two months were low. The committee decided to launch a week long campaign to promote fruits in each cafeteria which boosted their sales.

Benefits and challenges of stakeholder engagement

We are confident that our project, the project goals, methods and intervention have evolved positively based on our collaborations with stakeholders. Most importantly, their input was necessary in determining and describing cafeteria intervention. Their continuous involvement in monitoring helped in solving problems promptly and enhanced adherence to intervention. In addition, their input shaped methods, plans and processes for recruiting participants and data collection.

While we had a positive experience of involving stakeholder, it was not devoid of challenges. A major challenge was time availability of stakeholders as they were busy in their respective works. To address this, we scheduled meetings according to the availability of the stakeholders. We had to conduct multiple workshops and information sessions to accommodate the time of cafeteria staff and hospital staff. This was time and resource consuming. It was also important to allow time for stakeholders to get acquainted with their new roles associated with intervention. Their knowledge of a healthy diet was dominated by the cultural norms and there was some misconception. So, we invested time and resources in training them on healthy eating. This also led to initiation of positive changes in cafeterias during the control period before the predetermined intervention date. We also felt that research priorities do not necessarily meet the priorities of the stakeholders. For example, the cafeteria staff emphasized more on the changes in physical structures such as adding culinary. There was language barrier, as researchers were comfortable using scientific jargons such as 'epidemiology' 'control' 'confounding', 'subjects' etc. making the stakeholders confused at times. It was difficult to translate or interpret these works well in local language. Additionally, the food beliefs of researchers and stakeholders were different. Stakeholders strongly believe in eating

warm ‘white rice’ twice a day. In addition, fruits were not considered appropriate to take during lunch. Knowledge sessions on healthy diets addressed these deep rooted beliefs to some extent but changing food belief system takes longer time and more effort. [6,20]

DISCUSSION

We identified employees, cafeteria managers, workplace administrators, finance manager, human resource manager, cafeteria staff and researchers as major stakeholders in the Nepal pioneer worksite intervention study to prevent cardiometabolic risks. The stakeholders were included in the study team and they brought their expertise in the subject matter as well as insights as employees and customers of the cafeteria. The exploration of the facilitators and barriers to healthy eating at their worksite provided a base for defining the intervention and study attributes. Interactive workshops provided opportunity and mandate for the stakeholders to participate in decision making and strengthened the investigator-stakeholder relationship. Individual consultations helped get stronger buy-in and commitment from the higher authorities and employees. Regular meeting of the study team and canteen intervention committee guaranteed continuous engagement in problem solving and decision making. It reinforced ownership and enhanced project sustainability. The major challenges were the time-consuming nature of the process, language barriers, and differences in health and food beliefs between researchers and stakeholders.

We mapped and engaged 7 different types of stakeholders in our study. Patient-centered outcome research projects have reported engaging clinicians, caregivers, advocacy organizations and health system representatives; but only few engaged payers and policy makers[13,21]. We incorporated the views of multiple types of stakeholders into our study during formative phase, development of

intervention and study and execution of the study. For example, we formed cafeteria intervention committee based on the suggestion of cafeteria manager in in-depth interview. Brown-rice tasting experiment was also conducted to solve the concern of consumers and chefs on the acceptability of brown rice in workshop. Engagement has most frequently been reported in developing research questions[9]. This study described the effort, attention, resources and flexibility for concerted engagement emphasizing on meaningful and continuous collaboration, shared leadership and decision-making power, and adaptation to the needs of stakeholders. Other researchers have highlighted the importance of sharing power for successful research engagement[22].

The input from different stakeholder influenced our study goals, intervention, methods and materials. This enhanced the appropriateness and relevance in the local context. Similar experience was reported by other investigators[6]. Making research more beneficiaries-centric, culturally relevant and accessible to potential research participants have been reported to refine proposals, recruitment materials, and participant compensation[23]. –In our project, the stakeholders contributed to the interpretation of qualitative data and contextualize the findings, which was also reported by otherssimilar to the study by Boote J et al researc hers[11]. This is consistent with the available evidence on positive impacts of engaged research[6,12].

The stakeholder engagement has contributed positively in our study from formative phase to execution. Various levels of stakeholder involvement, such as consultation, collaboration or user-control has been proposed[24]. However, caution has been recommended against assuming that involving higher levels is better[25]. Some researchers in a national survey from UK claimed that decisions about the appropriateness of consumer involvement in research should be made on a case-by-case basis [13].

The major challenges that we faced were the time-consuming nature of the process, language barriers, and differences in health and food beliefs between researchers and stakeholders. Similar challenges have been reported in other studies[6,20]. Lack of time—both for stakeholders and researchers—was the most frequently noted challenge and was identified as a top barrier to engagement in a recent survey of investigators[20]. Thus, it is crucial to develop strategies that maximize stakeholder input with minimum time and resources[26].

The generalizability of our findings is limited because of the unique setting where the research was conducted. Because it was a university hospital, we had an advantage of including the employees with specific research expertise as co-investigators. The stakeholders had more interest in and experience with the health interventions and research. Thus, their knowledge and attitude about engagement may be more positive.

CONCLUSION

It was possible to engage and benefit from stakeholder's engagement on the design, implementation and monitoring of a workplace-based hypertension and diabetes management research program in Nepal. We recommend ongoing program development and implementation by stakeholder input. In future, we plan to evaluate the influence of stakeholders on the research project and assess the engagement and effectiveness through their perspective, as well as longer term outcomes on health decision making beyond the conduction of research.

ACKNOWLEDGMENTS

We would like to acknowledge the Dhulikhel Hospital – Kathmandu University Hospital for their permission and cooperation to conduct this study. We appreciate the contribution of all the study participants.

AUTHOR CONTRIBUTIONS

Conceptualization: Archana Shrestha (AS), Dipesh Tamrakar(DT), Donna Spiegelman (DS)

Formal analysis: Bhawana Shrestha (BS), Biraj Man Karmacharya(BMK), Abha Shrestha (AbS), Prajjwal Pyakurel(PP)

Writing – original draft: Archana Shrestha (AS)

Writing – review & editing: Archana Shrestha (AS), Dipesh Tamrakar (DT), Bhawana Shrestha (BS), Biraj Man Karmacharya(BMK), Abha Shrestha(AbS), Prajjwal Pyakurel(PP), Donna Spiegelman (DS)

REFERENCES

1. Titler MG. The Evidence for Evidence-Based Practice Implementation. In: Hughes RG, editor. Patient Safety and Quality: An Evidence-Based Handbook for Nurses. Rockville (MD): Agency for Healthcare Research and Quality (US); 2008. Available: http://www.ncbi.nlm.nih.gov/books/NBK2659/

2. Zilberberg MD. The clinical research enterprise: time to change course? Jama. 2011;305: 604–605.

3. Ray KN, Miller E. Strengthening stakeholder-engaged research and research on stakeholder engagement. Journal of comparative effectiveness research. 2017;6: 375–389.

4. Mathur VN, Price ADF, Austin S. Conceptualizing stakeholder engagement in the context of sustainability and its assessment. Construction Management and Economics. 2008;26: 601–609. doi:10.1080/01446190802061233

5. Kok MO, Gyapong JO, Wolffers I, Ofori-Adjei D, Ruitenberg J. Which health research gets used and why? An empirical analysis of 30 cases. Health research policy and systems. 2016;14: 1–18.

6. Shippee ND, Domecq Garces JP, Prutsky Lopez GJ, Wang Z, Elraiyah TA, Nabhan M, et al. Patient and service user engagement in research: a systematic review and synthesized framework. Health Expectations. 2015;18: 1151–1166.

7. Mullins CD, Abdulhalim AM, Lavallee DC. Continuous patient engagement in comparative effectiveness research. Jama. 2012;307: 1587–1588.

8. Lowes L, Hulatt I. Involving service users in health and social care research. Routledge; 2013.

9. Concannon TW, Fuster M, Saunders T, Patel K, Wong JB, Leslie LK, et al. A systematic review of stakeholder engagement in comparative effectiveness and patient-centered outcomes research. Journal of general internal medicine. 2014;29: 1692–1701.

10. Forsythe LP, Szydlowski V, Murad MH, Ip S, Wang Z, Elraiyah TA, et al. A systematic review of approaches for engaging patients for research on rare diseases. Journal of general internal medicine. 2014;29: 788–800.

11. Boote J, Baird W, Sutton A. Involving the public in systematic reviews: a narrative review of organizational approaches and eight case examples. Journal of comparative effectiveness research. 2012;1: 409–420.

12. Brett J, Staniszewska S, Mockford C, Herron-Marx S, Hughes J, Tysall C, et al. Mapping the impact of patient and public involvement on health and social care research: a systematic review. Health expectations. 2014;17: 637–650.

13. Barber R, Boote JD, Cooper CL. Involving consumers successfully in NHS research: a national survey. Health Expectations. 2007;10: 380–391.

14. Shrestha A, Tamrakar D, Karmacharya BM, Shrestha A, Shrestha R, Bhatta RD, et al. Nepal Pioneer Worksite Intervention Study to lower cardio-metabolic risk factors: design and protocol. BMC cardiovascular disorders. 2019;19: 1–10.

15. Concannon TW, Meissner P, Grunbaum JA, McElwee N, Guise J-M, Santa J, et al. A new taxonomy for stakeholder engagement in patient-centered outcomes research. Journal of general internal medicine. 2012;27: 985–991.

16. Boyatzis RE. Transforming qualitative information: Thematic analysis and code development. sage; 1998.

17. Gyawali P, Tamrakar D, Shrestha A, Shrestha H, Karmacharya S, Bhattarai S, et al. Consumer acceptance and preference for brown rice—A mixed-method qualitative study from Nepal. Food Science & Nutrition. 2022; fsn3.2803. doi:10.1002/fsn3.2803

18. Sciences NA of, Science NA of E (US) and I of M (US) C on, Engineering, Policy and P. ADVISING AND MENTORING. On Being a Scientist: A Guide to Responsible Conduct in Research: Third Edition. National Academies Press (US); 2009. Available: https://www.ncbi.nlm.nih.gov/books/NBK214555/

19. Tamrakar D, Shrestha A, Rai A, Karmacharya BM, Malik V, Mattei J, et al. Drivers of healthy eating in a workplace in Nepal: a qualitative study. BMJ Open. 2020;10: e031404. doi:10.1136/bmjopen-2019-031404

20. Boote J, Baird W, Beecroft C. Public involvement at the design stage of primary health research: a narrative review of case examples. Health policy. 2010;95: 10–23.

21. Forsythe LP, Ellis LE, Edmundson L, Sabharwal R, Rein A, Konopka K, et al. Patient and stakeholder engagement in the PCORI pilot projects: description and lessons learned. Journal of general internal medicine. 2016;31: 13–21.

22. Westfall JM, Fagnan LJ, Handley M, Salsberg J, McGinnis P, Zittleman LK, et al. Practice-based research is community engagement. The Journal of the American Board of Family Medicine. 2009;22: 423–427.

23. Joosten YA, Israel TL, Williams NA, Boone LR, Schlundt DG, Mouton CP, et al. Community engagement studios: a structured approach to obtaining meaningful input from stakeholders to inform research. Academic Medicine. 2015;90: 1646.

24. Dent H. Involving the Public in NHS, Public Health and Social Care Research: Briefing notes for researchers. Nurse Researcher. 2005;12: 89–91.

25. Barnes M, Cotterell P. Critical and different perspectives on user involvement. Critical perspectives on user involvement. 2012; 225–33.

26. Frank L, Forsythe L, Ellis L, Schrandt S, Sheridan S, Gerson J, et al. Conceptual and practical foundations of patient engagement in research at the patient-centered outcomes research institute. Quality of Life Research. 2015;24: 1033–1041.

1. Joyce MC. How will I ever keep up with pharmacy changes? Pharmacy Today. 2015;21: 1.

2. Zilberberg MD. The clinical research enterprise: time to change course? Jama. 2011;305: 604–605.

3. Ray KN, Miller E. Strengthening stakeholder-engaged research and research on stakeholder engagement. Journal of comparative effectiveness research. 2017;6: 375–389.

4. Mathur VN, Price ADF, Austin S. Conceptualizing stakeholder engagement in the context of sustainability and its assessment. Construction Management and Economics. 2008;26: 601–609. doi:10.1080/01446190802061233

5. Kok MO, Gyapong JO, Wolffers I, Ofori-Adjei D, Ruitenberg J. Which health research gets used and why? An empirical analysis of 30 cases. Health research policy and systems. 2016;14: 1–18.

6. Shippee ND, Domecq Garces JP, Prutsky Lopez GJ, Wang Z, Elraiyah TA, Nabhan M, et al. Patient and service user engagement in research: a systematic review and synthesized framework. Health Expectations. 2015;18: 1151–1166.

7. Mullins CD, Abdulhalim AM, Lavallee DC. Continuous patient engagement in comparative effectiveness research. Jama. 2012;307: 1587–1588.

8. Lowes L, Hulatt I. Involving service users in health and social care research. Routledge; 2013.

9. Concannon TW, Fuster M, Saunders T, Patel K, Wong JB, Leslie LK, et al. A systematic review of stakeholder engagement in comparative effectiveness and patient-centered outcomes research. Journal of general internal medicine. 2014;29: 1692–1701.

10. Forsythe LP, Szydlowski V, Murad MH, Ip S, Wang Z, Elraiyah TA, et al. A systematic review of approaches for engaging patients for research on rare diseases. Journal of general internal medicine. 2014;29: 788–800.

11. Boote J, Baird W, Sutton A. Involving the public in systematic reviews: a narrative review of organizational approaches and eight case examples. Journal of comparative effectiveness research. 2012;1: 409–420.

12. Brett J, Staniszewska S, Mockford C, Herron- Marx S, Hughes J, Tysall C, et al. Mapping the impact of patient and public involvement on health and social care research: a systematic review. Health expectations. 2014;17: 637–650.

13. Barber R, Boote JD, Cooper CL. Involving consumers successfully in NHS research: a national survey. Health Expectations. 2007;10: 380–391.

14. Shrestha A, Tamrakar D, Karmacharya BM, Shrestha A, Shrestha R, Bhatta RD, et al. Nepal Pioneer Worksite Intervention Study to lower cardio-metabolic risk factors: design and protocol. BMC cardiovascular disorders. 2019;19: 1–10.

15. Concannon TW, Meissner P, Grunbaum JA, McElwee N, Guise J-M, Santa J, et al. A new taxonomy for stakeholder engagement in patient-centered outcomes research. Journal of general internal medicine. 2012;27: 985–991.

16. Boyatzis RE. Transforming qualitative information: Thematic analysis and code development. sage; 1998.

17. Gyawali P, Tamrakar D, Shrestha A, Shrestha H, Karmacharya S, Bhattarai S, et al. Consumer acceptance and preference for brown rice—A mixed- method qualitative study from Nepal. Food Science & Nutrition. 2022; fsn3.2803. doi:10.1002/fsn3.2803

18. Sciences NA of, Science NA of E (US) and I of M (US) C on, Engineering, Policy and P. ADVISING AND MENTORING. On Being a Scientist: A Guide to Responsible Conduct in Research: Third Edition. National Academies Press (US); 2009. Available: https://www.ncbi.nlm.nih.gov/books/NBK214555/

19. Tamrakar D, Shrestha A, Rai A, Karmacharya BM, Malik V, Mattei J, et al. Drivers of healthy eating in a workplace in Nepal: a qualitative study. BMJ Open. 2020;10: e031404. doi:10.1136/bmjopen-2019-031404

20. Forsythe LP, Ellis LE, Edmundson L, Sabharwal R, Rein A, Konopka K, et al. Patient and stakeholder engagement in the PCORI pilot projects: description and lessons learned. Journal of general internal medicine. 2016;31: 13–21.

21. Westfall JM, Fagnan LJ, Handley M, Salsberg J, McGinnis P, Zittleman LK, et al. Practice-based research is community engagement. The Journal of the American Board of Family Medicine. 2009;22: 423–427.

22. Joosten YA, Israel TL, Williams NA, Boone LR, Schlundt DG, Mouton CP, et al. Community engagement studios: a structured approach to obtaining meaningful input from stakeholders to inform research. Academic Medicine. 2015;90: 1646.

23. Dent H. Involving the Public in NHS, Public Health and Social Care Research: Briefing notes for researchers. Nurse Researcher. 2005;12: 89–91.

24. Barnes M, Cotterell P. Critical and different perspectives on user involvement. Critical perspectives on user involvement. 2012; 225–33.

25. Boote J, Baird W, Beecroft C. Public involvement at the design stage of primary health research: a narrative review of case examples. Health policy. 2010;95: 10–23.

26. Frank L, Forsythe L, Ellis L, Schrandt S, Sheridan S, Gerson J, et al. Conceptual and practical foundations of patient engagement in research at the patient-centered outcomes research institute. Quality of Life Research. 2015;24: 1033–1041.

Response to Reviewers

08/22/2022

To

Editors and team

PLOS ONE journal

Subject: Re-submission of an original research article to PLOS ONE journal addressing the reviewer's comment

Dear sir/madam,

It is our pleasure to re-submit our manuscript entitled "**Stakeholder Engagement in a Hypertension and Diabetes Prevention Research Program**" to the editorial teams. The paper aims to describe the stakeholder engagement process and the lessons learned from this process while designing, implementing and monitoring a study on diabetes and hypertension prevention in workplace settings in Nepal. It provides important insights for future researchers that aim to engage stakeholders in national-level preventive programs in health.

We would like to thank all the reviewers for their constructive comments. The responses to the reviewer's comments are addressed individually below:

Reviewer 2

General:

An important topic that will contribute to stakeholder engagement in interventions knowledge base. But unless, I have not understood the title "It is description & lessons

learnt" As a reader I don't think I still can clearly pinpoint the lessons learnt unless in the manuscript it is equivalent to benefits & challenges. Wonder if the title should not be Description & experiences?

>> Based on your suggestions we removed Description & experiences from title.

Generally, well written. However, the manuscript can benefit from some editing e.g. Line 143: word interview should not be started with upper case. Line 144: Add "a" between guide and in private room. More information on specific places to correct are given in comments on the manuscript itself.

>>Edited based on your comments

I see some of the work mentioned in the manuscript has been published, for example the FDGs & in-depth interviews. I think it is important that the reader understands from the word go, that details are reported somewhere. For example, I had a question on the number of participants per FDG, and wondering about the participant's quotes, but I see these details are in reference 19.

>> We have added brief of the methods and results

I think what would important is to highlight what informed the authors decisions- for example to form a study committee, use focus groups/workshops and not any methods for engagement, in case a reader would want to conduct a similar stakeholder engagement but still briefly report how these activities were done. I believe you were guided by literature.

>> It is mentioned in under Engagement objectives heading.

2 Introduction: The statement "New scientific evidence" appears incomplete without saying what kind of evidence. For example, is it health promotion/medical intervention effectiveness?

>> Addressed

Methods:

Lines 122- 126: The formation of the study team is unclear. I would believe some stakeholder categories had more than one person. How did you decide which representative(s) to approach? Are there people who declined to take part, since you have said those who provided informed consent? The last part "The stakeholders represented as co-investigators and advisors" need to be rephrased for clarity. [Probably referring to your published work could help as well]. I think its important to mention the rationale for the constituents of study team in the methods section

>> The formation of the study team is now rephrased and edited to make more clear statement

Workshops: As a reader I will be interested to know the aim of the workshop. It may be good to give the ethics approval number The authors should detail how stakeholders were involved in the interpretation of the results.

>>Aim of workshop and approval number are added in the manuscript

Line 352- put a reference as this is documented in the literature. I believe the first part of the discussion i.e. 356 to 367, there would be literature documenting these strategies in other studies. If not the authors should indicate there is no other studies identified that have used the same strategies as the current study.

>>Reference is added to the line 352.

Conclusion does not include any comment on lessons learnt.

>> We removed lesson learnt from title. And hence lesson learnt is not placed in the conclusion section.

We look forward to your comments and remain with best regards;

Yours sincerely,

Dr. Archana Shrestha

Department of Public Health

Kathmandu University School of Medical Sciences

Dhulikhel, Nepal
