# Supplementary material for: Stakeholder engagement in a hypertension and diabetes prevention research program: Description and lessons learned
Source: PLoS One. 2022 Oct 20;17(10):e0276478. doi: 10.1371/journal.pone.0276478 (PMC9584412; doi:10.1371/journal.pone.0276478)
Supplement: Attachment — Submitted filename: Response to Reviewers.docx [file pone.0276478.s003.docx]

**Response to Reviewers**

**11/05/2022**

**To**

**Editors and team**

**PLOS ONE journal**

**Subject: Re-submission of an original research article to PLOS ONE journal addressing the reviewer's comment**

Dear sir/madam,

It is our pleasure to re-submit to you and the editorial teams our manuscript entitled “**Stakeholder Engagement in a Hypertension and Diabetes Prevention Research Program – Description and Lessons Learned**”. The paper aims to describe the stakeholder engagement process and the lessons learned from this process while designing, implementing and monitoring a study on diabetes and hypertension prevention in workplace settings in Nepal. It provides important insights for future researchers that aim to engage stakeholders in national-level preventive programs in health.

We would like to thank all the reviewers for their constructive comments. The responses to the reviewer’s comments are addressed individually below:

**Reviewer 1**

This manuscript describes stakeholder engagement activities and lessons learned in diabetes and hypertension prevention study in workplace settings in Nepal. The authors describe stakeholder engagement activities during the formative, intervention development, and study conduct phases of this project. In general, this paper could benefit from a grammatical review as there are several errors throughout. Additionally, the literature review should be updated to reflect current gaps, as there are several existing articles to date that offer engagement implementation examples, which was stated as a gap and strength of current paper. The impact of stakeholder engagement on research activities and translation is an area for further contribution and an opportunity where this manuscript could offer insight. Areas of stakeholder impact should be more clearly identified in the paper. In addition to above, consider the following to strengthen this manuscript:

-Consider re-designing Table 1 into a Figure and move to first presentation of stakeholders using 7P Framework in Methods section (not in Results section as current placement).

***>> The Table 1 is converted to figure and shifted to methods section***

-Although it was stated that results from qualitative work is under consideration for publication elsewhere, this section could benefit from including more specifics regarding questions asked during focus group sessions/in-depth interviews with stakeholders and/or qualitative supporting quotes from discussions, since these informed intervention. Who came up with interview questions? How did the research team decide which feedback to implement?

*>> T****he result and methods are available in detailed in the paper below and it is now cited.***

***Tamrakar D, Shrestha A, Rai A, Karmacharya BM, Malik V, Mattei J, et al. Drivers of healthy eating in a workplace in Nepal: a qualitative study. BMJ Open. 2020;10: e031404. doi:10.1136/bmjopen-2019-031404***

-Consider creating a new Table 1 in Results that includes stakeholder type and the actual activities that the stakeholders were engaged in (i.e. formative and then subheading for which activity i.e. focus group). It was not clear within text the myriad of stakeholders who participated in the various study processes. This table could also include a column "Impact" to highlight how stakeholder contributions informed the study and in what way(s).

***>>Table is generated as per your suggestions***

**Reviewer 2**

An important work in the area stakeholder engagement. Generally, well written but needs some language editing and improvement in flow in some sections.

*Abstract*

Lines 39-41. What is described does not include interpreting the results and results dissemination.

***>> It is revised as per your suggestion***

Lines 47: I am not sure increased trust in research and confidence with the researchers is a result or what can be inferred (or assumed) given the processes and outcomes. What evidence do you have for this allusion?

***>> It is revised as per your suggestion***

Line 54: The manuscript has not reported on engaging the stakeholders in the evaluation may be monitoring.

***>>The comment is addressed***

Introduction

Line 62: Give reference

***>> Reference is added***

See comment on line 67

***>> comment addressed***

Materials and methods

-Line 88: Aim would be more appropriate than “theme”

***>> “theme” is changed to “aim”***

-Sub-titles e.g., Phase 1 Formative study would be better be presented as Phase 1: Formative study

-Lines 106 to lines 202: Flow in stakeholder engagement needs to be improved for easier reading. I suggest a main sub-title “Stakeholder engagement” and smaller sub-titles as follows: stakeholder definition, engagement objectives, stakeholder identification, and stakeholder methods of engagement.

***>> Its flow is changed according to the suggestion provided.***

-Not sure it is necessary to number the phases e.g., line 134

***>>The phrases are now un-numbered***

-Lines 135-137. What criteria did you use to identify/include the individuals from each stakeholder category after conducting the mapping?. This also applies to those included in focus groups discussion (FDGs).

***>> Added to the manuscript. We selected adults aged 18 years or older, employee of the hospital, who provided informed consent in each stakeholder category.***

-Purposive selection for interviews- what specific information did you expect that these participants could be having unlike others

>> ***They had unique experience of making first hand decisions and implementation at the cafeteria and other wellness programs for the employees. These interviewees brought their perspectives on the decision making system, how it affected healthy food choices in the cafeteria and other administrative decisions regarding employee’s health. This would provide insights into the facilitators and barriers to healthy eating in the workplace environment.***

-I think its important to mention qualitative methods were used for FDGs and the in-depth interviews- as the methods hold specific benefits and give support from the literature on the choice of method

>> *>> T****he result and methods for FGDs and in depth interviews are available in detailed in the paper below and it is now cited.***

***Tamrakar D, Shrestha A, Rai A, Karmacharya BM, Malik V, Mattei J, et al. Drivers of healthy eating in a workplace in Nepal: a qualitative study. BMJ Open. 2020;10: e031404. doi:10.1136/bmjopen-2019-031404***

-Line 172: I think topic rather than theme will be more appropriate

***>> The word theme is replaced by the topic***

-What was the stakeholders’ role in the workshops?

*>>****The stakeholders provided their inputs in decision making. We presented the initial findings with the stakeholders and collected their feedback in the interpretation of the results.***

Results

-Line 211: What informed/guided the inclusion to a certain category (investigator vs. advisor). Any evidence from literature?

***>> Investigator were those who directly participated in the day-to-day implementation of the study, compared to advisors who provided their suggestions and guidances when asked for.***

-As a reader, I was expecting more details of the FDGs and interviews, but I saw at the end the statement that the results are under consideration for publication elsewhere. Just wondering, the implication of this given that data needs to be availed by authors.

>>*>> T****he result and methods are available in detailed in the paper below and it is now cited.***

***Tamrakar D, Shrestha A, Rai A, Karmacharya BM, Malik V, Mattei J, et al. Drivers of healthy eating in a workplace in Nepal: a qualitative study. BMJ Open. 2020;10: e031404. doi:10.1136/bmjopen-2019-031404***

-Lines 231-232: They suggested to add the healthy items which will demand less time and resources like adding the automated machines such as roti makers. Rephrase I don’t think automated machines needs less resource (may be human but not financial)

*>>* ***The sentence is rephrased as “They suggested to add the healthy items which will demand less time and human resources like adding the automated machines such as roti makers.”***

-Interchange order of sentences on lines 218-219 and lines 219-221 for better flow.

***>> The sentences are interchanged***

-What was the outcome of the food testing and how did it inform the intervention?

*>>* ***Added to the manuscript.*** ***Participant’s perspectives on brown rice was found to be changed after tasting. Participants were willing to switch to brown rice as they found that brown rice had higher quality.[17]***

-Rephrase lines 356-358 for clarity

***>>The line is rephrased for clarity***

-Lines 392-394: There is nowhere that is reported how the stakeholders were involved in interpreting the data.

***>> The comment is addressed in the manuscript.***

Discussion

Lines 379 to 387: It would be useful if the authors could highlight on how they incorporated the views of the stakeholders in each step, probably in a Table format. For example; What was the final goal and what stakeholder input lead to that outcome.

***>> We added some ways that we used to incorporate views of stakeholders. (. For example, we formed cafeteria intervention committee based on the suggestion of cafeteria manager in in-depth interview. Brown-rice tasting experiment was also conducted to solve the concern of consumers and chefs on the acceptability of brown rice in workshop.)***

***Other comments***

1. **Please ensure you have included the registration number for the clinical trial referenced in the manuscript.**

The registration number is added in the manuscript. This study was registered on clincaltrials.gov (Identification Member:[NCT03447340](https://clinicaltrials.gov/ct2/show/NCT03447340); Registration date: 27th February, 2018).

2. **To help ensure that the wording of your manuscript is suitable for publication, would you please also add this statement at the beginning of the Methods section of your manuscript file.**

The ethical statement is added in the publication.

**Ethical consideration:** We received approval from the institutional review board of Kathmandu University School of Medical Sciences and Nepal Health Research Council for the human subject contacts and obtained written informed consent from the participants before collecting data for research purpose.

3. Data availability statement.

The dataset is uploaded is OSF. The link to dataset is https://osf.io/bqsrd/

I think it is important for the reader to know where the project is at the moment- is still being implemented and prospect of sustainability given that its one of the objective of involving stakeholders

We look forward to your comments and remain with best regards;

**Yours sincerely,**

**Dr. Archana Shrestha**

Department of Public Health

Kathmandu University School of Medical Sciences

Dhulikhel, Nepal
