# Supplementary material for: Stakeholder engagement in a hypertension and diabetes prevention research program: Description and lessons learned
Source: PLoS One. 2022 Oct 20;17(10):e0276478. doi: 10.1371/journal.pone.0276478 (PMC9584412; doi:10.1371/journal.pone.0276478)
Supplement: Attachment — Submitted filename: Response to Reviewers.docx [file pone.0276478.s005.docx]

**Response to Reviewers**

**08/22/2022**

**To**

**Editors and team**

**PLOS ONE journal**

**Subject: Re-submission of an original research article to PLOS ONE journal addressing the reviewer's comment**

Dear sir/madam,

It is our pleasure to re-submit our manuscript entitled “**Stakeholder Engagement in a Hypertension and Diabetes Prevention Research Program**” to the editorial teams. The paper aims to describe the stakeholder engagement process and the lessons learned from this process while designing, implementing and monitoring a study on diabetes and hypertension prevention in workplace settings in Nepal. It provides important insights for future researchers that aim to engage stakeholders in national-level preventive programs in health.

We would like to thank all the reviewers for their constructive comments. The responses to the reviewer’s comments are addressed individually below:

**Reviewer 2**

General:

**An important topic that will contribute to stakeholder engagement in interventions knowledge base. But unless, I have not understood the title “It is description & lessons learnt” As a reader I don’t think I still can clearly pinpoint the lessons learnt unless in the manuscript it is equivalent to benefits & challenges. Wonder if the title should not be Description & experiences?**

>> Based on your suggestions we removed Description & experiences from title.

**Generally, well written. However, the manuscript can benefit from some editing e.g. Line 143: word interview should not be started with upper case. Line 144: Add “a” between guide and in private room. More information on specific places to correct are given in comments on the manuscript itself.**

>>Edited based on your comments

**I see some of the work mentioned in the manuscript has been published, for example the FDGs & in-depth interviews. I think it is important that the reader understands from the word go, that details are reported somewhere. For example, I had a question on the number of participants per FDG, and wondering about the participant’s quotes, but I see these details are in reference 19.**

>> We have added brief of the methods and results

**I think what would important is to highlight what informed the authors decisions- for example to form a study committee, use focus groups/workshops and not any methods for engagement, in case a reader would want to conduct a similar stakeholder engagement but still briefly report how these activities were done. I believe you were guided by literature.**

>> It is mentioned in under Engagement objectives heading.

**2 Introduction: The statement “New scientific evidence” appears incomplete without saying what kind of evidence. For example, is it health promotion/medical intervention effectiveness?**

>> Addressed

Methods:

**Lines 122- 126: The formation of the study team is unclear. I would believe some stakeholder categories had more than one person. How did you decide which representative(s) to approach? Are there people who declined to take part, since you have said those who provided informed consent? The last part “The stakeholders represented as co-investigators and advisors” need to be rephrased for clarity. [Probably referring to your published work could help as well]. I think its important to mention the rationale for the constituents of study team in the methods section**

>> The formation of the study team is now rephrased and edited to make more clear statement

**Workshops: As a reader I will be interested to know the aim of the workshop. It may be good to give the ethics approval number The authors should detail how stakeholders were involved in the interpretation of the results.**

>>Aim of workshop and approval number are added in the manuscript

**Line 352- put a reference as this is documented in the literature. I believe the first part of the discussion i.e. 356 to 367, there would be literature documenting these strategies in other studies. If not the authors should indicate there is no other studies identified that have used the same strategies as the current study.**

>>Reference is added to the line 352.

**Conclusion does not include any comment on lessons learnt.**

>> We removed lesson learnt from title. And hence lesson learnt is not placed in the conclusion section.

We look forward to your comments and remain with best regards;

**Yours sincerely,**

**Dr. Archana Shrestha**

Department of Public Health

Kathmandu University School of Medical Sciences

Dhulikhel, Nepal
