# Supplementary material for: Stakeholder engagement in a hypertension and diabetes prevention research program: Description and lessons learned
Source: PLoS One. 2022 Oct 20;17(10):e0276478. doi: 10.1371/journal.pone.0276478 (PMC9584412; doi:10.1371/journal.pone.0276478)
Supplement: Attachment — Submitted filename: Response to Reviewers.docx [file pone.0276478.s007.docx]

**Response to Reviewers**

**29/09/2022**

**To**

**Editors and team**

**PLOS ONE journal**

**Subject: Re-submission of an original research article to PLOS ONE journal addressing the reviewer's comment**

Dear sir/madam,

It is our pleasure to re-submit to you and the editorial teams our manuscript entitled “**Stakeholder engagement in a hypertension and diabetes prevention research program: description and lessons learned**”. The paper aims to describe the stakeholder engagement process and the lessons learned from this process while designing, implementing and monitoring a study on diabetes and hypertension prevention in workplace settings in Nepal. It provides important insights for future researchers that aim to engage stakeholders in national-level preventive programs in health.

We would like to thank all the reviewers for their constructive comments. All comments are addressed and the track-changed files along with the clean manuscript files are submitted to the journal.

We look forward to your comments and remain with best regards.

**Yours sincerely,**

**Dr. Archana Shrestha**

Department of Public Health

Kathmandu University School of Medical Sciences

Dhulikhel, Nepal
